# Cellular Tracking and Gene Profiling of *Fusarium graminearum* during Maize Stalk Rot Disease Development Elucidates Its Strategies in Confronting Phosphorus Limitation in the Host Apoplast

**DOI:** 10.1371/journal.ppat.1005485

**Published:** 2016-03-14

**Authors:** Yan Zhang, Juan He, Lei-Jie Jia, Ting-Lu Yuan, Dong Zhang, Yan Guo, Yufeng Wang, Wei-Hua Tang

**Affiliations:** 1 National Key Laboratory of Plant Molecular Genetics, Center for Excellence in Molecular Plant Sciences, Institute of Plant Physiology and Ecology, Shanghai Institutes for Biological Sciences, Chinese Academy of Sciences, Shanghai, China; 2 Department of Biology, South Texas Center for Emerging Infectious Diseases, University of Texas at San Antonio, San Antonio, Texas, United States of America; Wageningen University, NETHERLANDS

## Abstract

The ascomycete fungus *Fusarium graminearum* causes stalk rot in maize. We tracked this pathogen’s growth in wound-inoculated maize stalks using a fluorescence-labeled fungal isolate and observed that invasive hyphae grew intercellularly up to 24 h post inoculation, grew intra- and inter-cellularly between 36–48 h, and fully occupied invaded cells after 72 h. Using laser microdissection and microarray analysis, we profiled changes in global gene expression during pathogen growth inside pith tissues of maize stalk from 12 h to six days after inoculation and documented transcriptomic patterns that provide further insights into the infection process. Expression changes in transcripts encoding various plant cell wall degrading enzymes appeared to correlate with inter- and intracellular hyphal growth. Genes associated with 36 secondary metabolite biosynthesis clusters were expressed. Expression of several *F*. *graminearum* genes potentially involved in mobilization of the storage lipid triacylglycerol and phosphorus-free lipid biosynthesis were induced during early infection time points, and deletion of these genes caused reduction of virulence in maize stalk. Furthermore, we demonstrated that the *F*. *graminearum* betaine lipid synthase 1 (BTA1) gene was necessary and sufficient for production of phosphorus-free membrane lipids, and that deletion of *BTA1* interfered with *F*. *graminearum’s* ability to advance intercellularly. We conclude that *F*. *graminearum* produces phosphorus-free membrane lipids to adapt to a phosphate-limited extracellular microenvironment during early stages of its invasion of maize stalk.

## Author Summary

*Gibberella* stalk rot of maize is an economically important crop disease caused by the filamentous fungus *Fusarium graminearum* (previously also called *Gibberella zeae*). Stalk rots develop in the main stem of mature maize plants. Detailed anatomical studies of the complete infection process in maize stalk have not previously been reported at the cellular level. Using a fluorescently-tagged strain of *F*. *graminearum*, we observed that the fungus initially grows between live maize cells (intercellular growth) and only later penetrates host cells, also killing nearby plant cells. Using laser microdissection, we selectively retrieved fungal biomass from infected maize stalks and obtained high-resolution gene expression profiles for the fungal-enriched samples at 0, 12, 18, 36, 48, 72, 108, 132 and 144 hours after inoculation (hai). Many of the gene expression patterns are consistent with microscopically observed fungal growth at the corresponding stage of infection. For example, enzymes with the potential to break pectin primary chains are most highly expressed at 12–36 hai when *F*. *graminearum* is observed to grow, for the most part, intercellularly, for which breakdown of pectin, a major intercellular component, is required. We propose a previously unreported strategy for the successful colonization of maize stalk by *F*. *graminearum* that involves the enzyme betaine lipid synthase 1: the pathogen produces phosphorus-free membrane lipids which allows it to maintain an aggressive intercellular advance in the phosphorus depleted environment of the maize apoplast before it kills nearby plant cells and releases their cellular nutrients.

## Introduction

The main stem (i.e. stalk) of maize (*Zea mays*) represents about 50% of the total plant dry biomass at the grain maturity stage [1]. Stalk rot is a major disease of maize worldwide [2], and tends to be more common in higher yielding hybrids that produce larger ears [3]. The ascomycete fungal genus *Fusarium* is the most frequently reported causative agent of maize stalk rot diseases [3]. *Fusarium graminearum* (previously also named *Gibberella zeae*), which can cause *Gibberella* stalk rot, is thought to possess the highest pathogenicity and aggressiveness among species responsible for stalk rot [4]. No specific resistance genes that confer immunity to this disease have been identified to date. A few quantitative trait loci for resistance have been reported, but the nature of the resistance still unknown [5, 6]. The diferulic acid content of the cell walls of maize stalks has been linked to quantitative resistance by an inbred lines survey [4]. The molecular mechanisms underlying fungal pathogenesis during maize stalk infection are not clear, which limits progress toward effectively controlling the disease.

The final symptom of *Gibberella* stalk rot is lodging (i.e. breakage of the stalk below the ear), which can result in loss of harvestable yield. In the late stages of infection, small, round, black specks (perithecia, the sexual structures of *F*. *graminearum*) are formed on the surface of the stalk rind. The stalks become hollow tubes with only the rinds and vascular bundle strands remaining. *F*. *graminearum* may enter the host through the roots of seedlings or through the bases of the leaf sheaths of young plants, where spores (sexual ascospores or asexual conidia) may germinate and grow up into the stalk. Spores may also directly enter adult maize stems through wounds made by corn borers, hail, or mechanical injury [7]. How this fungus manages to overcome various physical barriers and the active defenses of the host and progressively accommodates itself inside the stalk is largely unknown.

Upon *F*. *graminearum* infection, maize stalk produces kauralexins and zealexins, which can inhibit fungal growth *in vitro* [8–10]. The invasion of living tissues requires the fungus to be able to break barriers (such as plant cell walls and plasma membranes), and to overcome active host defenses which often involve reactive oxygen species, anti-fungal proteins and small molecular weight metabolites like the phytoalexins mentioned above) [11, 12]. The *F*. *graminearum* genome has been sequenced [13] and the current annotation [14] reveals an arsenal of potential invasion-related factors including secreted plant cell wall degrading enzymes, proteases, secondary metabolites (including mycotoxins) biosynthesis gene clusters, and so on [15]. But how *F*. *graminearum* chooses from this arsenal to confront maize stalk defenses is yet to be discovered.

Besides causing stalk rot of maize, *F*. *graminearum* also causes Fusarium head blight on wheat and barley, ear rot disease of maize, and seedling blight on maize and wheat [16–18]. It has been reported that *F*. *graminearum* forms a specialized infection structure on wheat florets [19], then grows inside the florets, expands through the rachis, and travels down to colonize the stem finally forming sexual structures known as perithecia [20]. The pathogenesis of wheat head blight caused by *F*. *graminearum* has been intensively studied. One key event in wheat head blight infection is the production of the harmful trichothecene mycotoxin deoxynivalenol (DON) [21]. DON binds to the peptidyltransferase of ribosomes and inhibits protein synthesis [22], allowing *F*. *graminearum* to pass through rachis and escape host cell wall thickening [23]. The outcomes of plant cell exposure to DON include hydrogen peroxide production, host programmed cell death and probably ethylene signaling [24–27], with specific details remaining to be elucidated. Interestingly, although DON is produced during barley head blight infection, it is not a virulence factor for barley head infection [23]. It has been reported that low amounts of DON can be detected in maize stalks from fields with stalk rot diseases, (much lower than the level detected in infected ears of maize [28]). The role of DON in maize stalk infection is still unknown. Other than DON production genes, numerous virulence genes have been identified in *F*. *graminearum* [29–31]. Because mutants of many virulence genes also showed severe defects in *in vitro* growth, these genes are considered as fungal essential genes. It is therefore more accepted to consider those dispensable for *in vitro* fungal growth but required for full virulence as pathogenicity genes. The number of pathogenicity genes using this definition is still limited, including genes encoding a secreted lipase FGL1 [32], a cyclin-dependent kinase CDC2A [33], two other type filamentous fungi-specific protein kinases (Fg03146 and Fg04770) [34], a Rab GTPase Rab2 [35], four phosphatases [36], transcription factors FGSG_01915 [37] and Tri6 [38], and a secondary metabolite biosynthesis cluster gene FGSG_10992 [39]. Most of the proteins encoded by pathogenicity genes are predicted to be intracellular proteins of *F*. *graminearum*, and thus are not in direct contact with plant components. Only FGL1 is an extracellular enzyme, mediating release of polyunsaturated free fatty acids that inhibit plant callose synthase activity [40]. Most of these Fusarium head blight pathogenicity genes have not been tested in maize stalk infection, except for CDC2A whose deletion also caused reduction in maize stalk infection [33]. It certainly will be interesting to delineate details of pathogenicity conservation and divergence between wheat head blight infection and maize stalk infection, two very different diseases both caused by the same fungus.

In the current study, we assessed fungal growth during maize stalk rot development at the cellular level, then used laser microdissection and microarray analysis to provide high-resolution gene expression profiles of *F*. *graminearum* transcripts over the course of infection at eight time points spanning from 12 h to 6 days after inoculation. A number of gene expression changes can be ascribed to invasive and/or survival strategies tailored to maize stalk infection. We also obtained evidence for non-phosphorus lipid accumulation during early infection. Using targeted gene knockout mutants, Using targeted gene knockout mutants, we show that a fungal betaine lipid synthase responsible for biosynthesis of non-phosphorus membrane lipids, two putative triacylglycerol lipases and three putative phosphatases are required for *F*. *graminearum* full virulence in maize stalk. We propose that *F*. *graminearum* employs a strategy to produce non-phosphorus membrane lipids in order to overcome growth-limiting phosphorus levels during intercellular growth in maize stalk.

## Results

### *Fusarium graminearum* hyphae first grow intercellularly and later intracellularly

**Fig 1:**
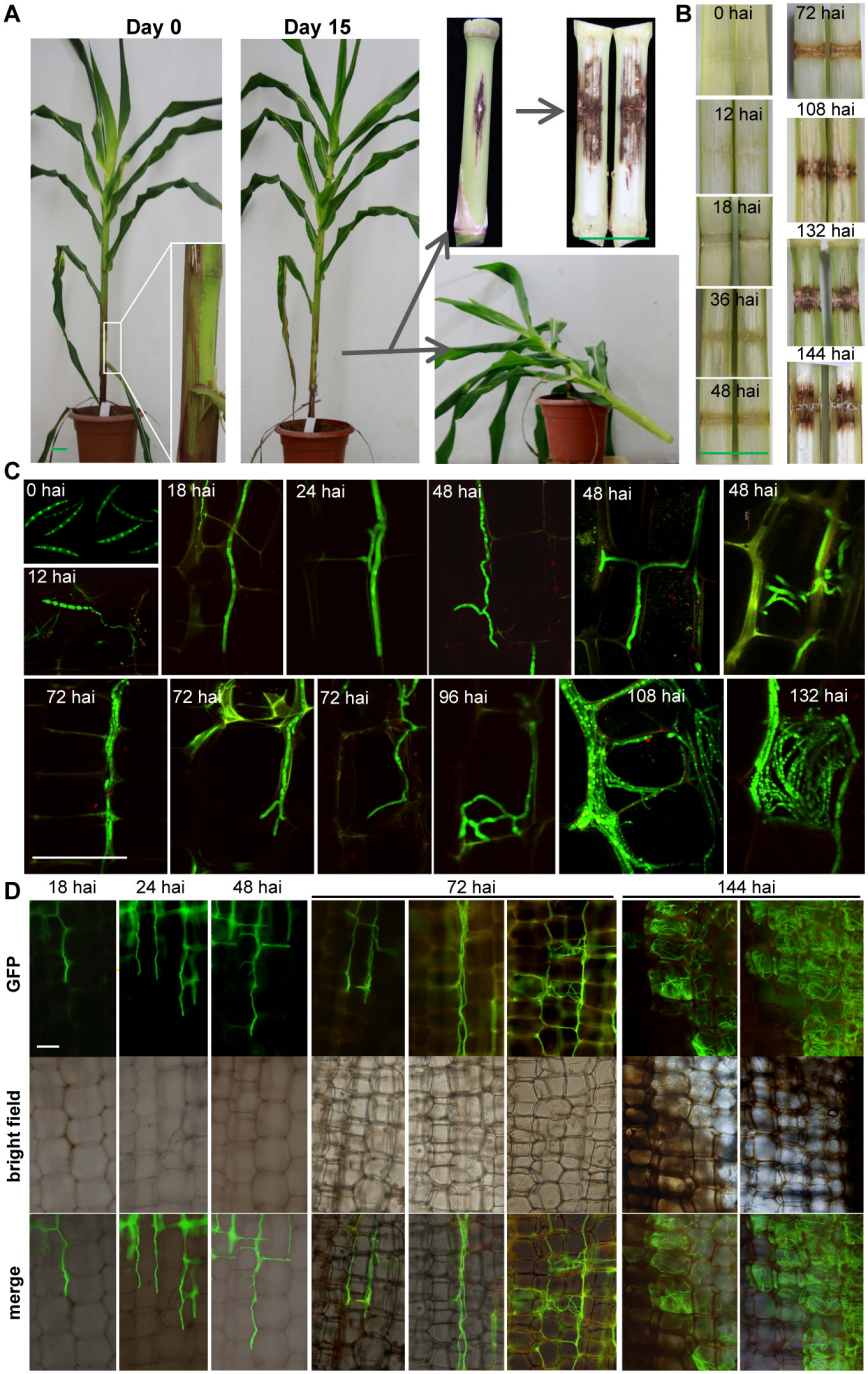
Tracking *Fusarium graminearum* growth in maize stalk. (A) The procedure from wound inoculation to split internodes ready for observation. (B) Representative split internodes at indicated time points. (C)-(D) Representative confocal (C) and wide-field (D) microscopic images of infected tissue from longitudinal sections. Maize plants were inoculated with spores of AmCyan-expressing *F*. *graminearum* (green under a GFP channel) and kept growing until the indicated timepoints when the stalk internodes were split and subjected to immediate microscopy. In this figure we focused on pith parenchyma cells; see Figure B in S1 Text for an uninfected reference, and Figure C in S1 Text for more pictures including rind areas. Green bar = 5 cm. White bar = 100 μm. hai: hours after inoculation.

To shed light on the process of *F*. *graminearum* proliferation inside maize stalks, we wounded the stalk of whole plants of maize roughly at the appearance of the tenth leaf, and inoculated plants with spores of a fluorescently(AmCyan)-tagged *F*. *graminearum* strain, which, other than its fluorescence, behaves like wild-type strains [39, 41]. From 2 h to 2 weeks after inoculation, we harvested individual maize plants, split the inoculated internodes and observed fungal migration and the appearance of the stalk tissue in detail (Fig 1A and Figure A in S1 Text).

To the naked eye, the split internodes were almost symptomless for up to 36 h after inoculation (hai). Stalk tissues at the wound site turned pale brown by 48 hai, and by 72 hai the brown color became darker and the brown area elongated above and below the site of the wound (Fig 1B).

We then observed the infection process by wide-field and confocal microscopy. By 12 hai, the fungal spore germinated, and then advanced inside stalk tissues in a hyphal form. In general, fungal hyphae grew longitudinally rather than transversely (Fig 1C and 1D). There are three major groups of cells in maize stalks, fiber cells in the rind, pith parenchyma cells, and vascular bundles (Figure B in S1 Text). Fungal hyphal progression in the pith precedes progression in the rind, but vascular bundles seemed to be avoided (Figure C in S1 Text).

**Fig 2:**
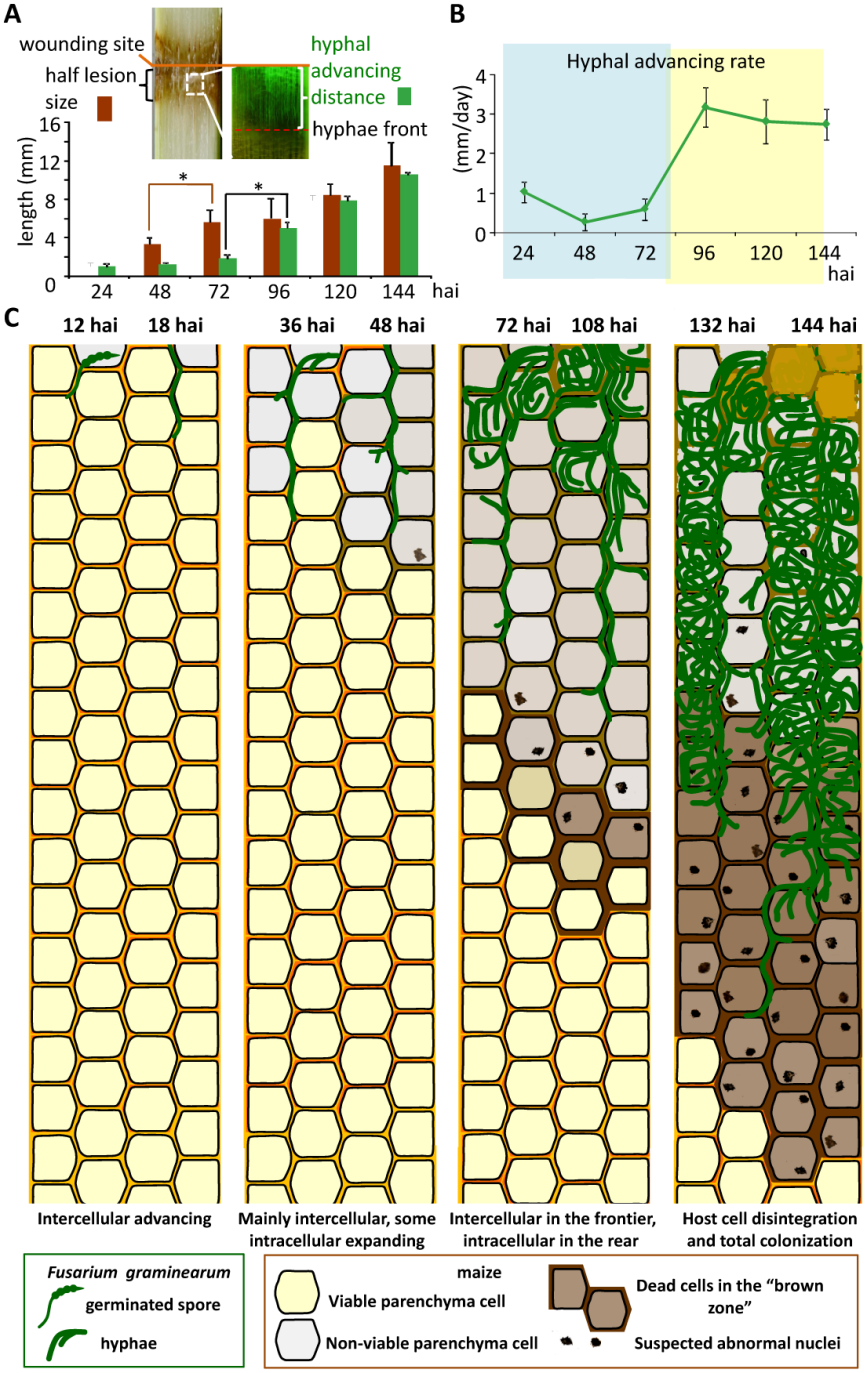
Measurements and diagram of maize stalk infection process. (A) Measurements of half lesion size and hyphal advance distances. Error bars indicate SD. *Significant difference by Student’s *t*-test (P < 0.05). Sample size n ≥3. (B) The advance rates of the hyphal front calculated based on the data in (A). (C) Diagram of cellular events during the advance of *F*. *graminearum* in stalk pith mainly composed of parenchyma cells. The top of each column represents the wound site. *F*. *graminearum* grew up and down in the stalk in a similar pattern and with a similar expansion speed; for simplicity only the downside from the wound site is shown. Cell viability was judged based on plasmolysis assay. hai: hours after inoculation.

**Fig 3:**
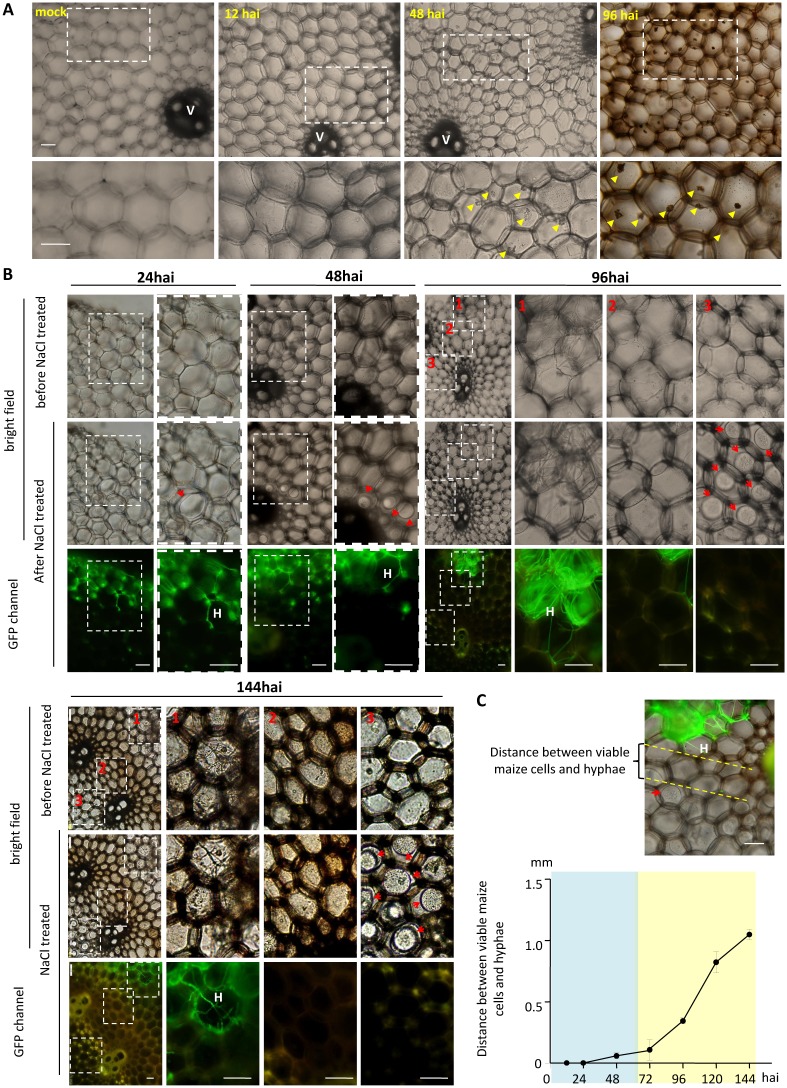
*graminearum* caused nuclear abnormalities and cell death in nearby maize cells from 48 hai. ***F***. (A) Representative bright field images of maize stalk pith tissues. Images 12, 48 and 96 hai were taken of the cross sections preceding the hyphal front (as indicated by the red broken line in Fig 2A). Yellow arrowheads point to nucleus-like structures. V: vascular bundle. (B) Representative images showing infected maize pith tissues before and after 1 M NaCl treatment for 10 min. *F*. *graminearum* AmCyanPH-1 hyphae are visible as green lines under a GFP channel. Red arrows point to maize cell membranes away from cell walls. H: hyphae. White scale bar = 100 μm. (C) The shortest distances between fungal hyphae and maize cells undergoing plasmolysis were measured. Error bars indicate SD. Sample size n ≥4. Images in this figure were all taken from cross sections. hai: hours after inoculation.

Fig 1C and Figure C in S1 text show that at 12–18 hai, most hyphae in the pith, which were unbranched and originated from germinated spores at wound sites, were found in the intercellular space of pith tissue, up to five layers of parenchyma cells away from the wounded layer, while the maize pith tissue remained symptomless to the naked eye. At approximately 18 hai, some multiple unbranched hyphae grew in parallel with each other along the intercellular space. At 24–36 hai, the majority of hyphae were unbranched and advanced intercellularly, but some hyphae had become branched and entered intracellular space. The stalk tissues still appeared symptomless under white light; but under UV light, the plant cell walls of an area beyond the fungal invasion exhibited strong autofluorescence (Figure D in S1 Text). At approximately 48 hai, more hyphae had penetrated maize cells, while the majority of hyphae were still intercellular. The infected pith tissues turned pale brown; the brown areas extended beyond the area hyphae had reached, and many vascular bundles far beyond the infected area turned brown. At approximately 72 hai, near the wounding site, some host parenchyma cell spaces were occupied by hyphae, while these parenchyma cell walls (marked by strong autofluorescence) still maintained a regular shape (Figure C in S1 Text). The hyphae at the advancing frontline continued to be intercellular. The brown area of plant tissues darkened, and also expanded, still exceeding the area reached by the fungal hyphae. The lengths of lesions increased most rapidly at 48–72 hai, preceding the peak time of the hyphal front extension, which occurred at 72–96 hai (Fig 2). Interestingly, by around 96 hai, most maize cells in the brown zone exhibited debris-like structures (Fig 3A). The structure could be weakly stained with 4’,6-diamino-2-phenylindole (DAPI) (Figure E in S1 Text) and we therefore suspected them to be deformed nuclei. It is worth noting that many maize cells that harbored deformed nuclei were not directly invaded by *F*. *graminearum* hyphae, but were a few cell layers away from those hyphae. At approximately 108 hai, the host cell walls within the infected area became deformed and partially disappeared (Figure C in S1 Text). At approximately 132–144 hai, most of the infected cells near the wound site were full of hyphae, and the pith tissue had totally collapsed (Figure C in S1 Text). The brown lesions further expanded to approximately 12 mm on either side of the wound site at 144 hai. After 360 hai, only the vascular strands remained intact, although they turned black, and the pith tissue in the center of the infected area had disintegrated.

Condensed or deformed nuclei can be seen in cells that undergo programmed cell death [42]. We assessed the viability of host cells near to fungal hyphae using a NaCl treatment (see Methods), and found that within 24 hai, among the maize cells that had been reached by hyphae, at least some still exhibited plasmolysis (Fig 3B and Figure F in S1 Text), suggesting these cells still had their plasma membrane intact and may thus be alive; around 48–72 hai, we did not observe plasmolysis by any maize cells that directly interacting with fungal hyphae, but maize cells adjacent to hyphal-invaded cells exhibited plasmolysis (Fig 3B and Figure F in S1 Text); around 96 hai, only those cells that were beyond or at the edge of the brown zone were still capable of plasmolysis (Fig 3C); around 144 hai, only cells beyond the brown zone maintained plasmolytic ability. Our results indicate that, at the early intercellular invasion stage, the region of dead host cells was limited to those directly interacting with fungal hyphae. Later, the “killing zone” extended to surrounding cells without direct interaction (Fig 3C).

In summary (Fig 2C), the proliferation of *F*. *graminearum* in maize stalk pith is at first exclusively a process of intercellular growth (12–18 hai). Then, intercellular growth continues along with the initiation of intracellular growth, in which the pathogen penetrates the surrounding parenchyma cells (24–48 hai) and then occupies the invaded parenchyma cells (72–108 hai), ultimately destroying the host pith tissues except for the vascular bundles (132–144 hai). In the early time points (12–48 hai), the fungal growth route is clearly restricted by plant cell wall structures, and at later time points (72–144 hai) there seems to be no plant restriction of fungal growth. Before 48 hai, the infected stalk tissue showed no symptoms visible to the unaided eye; after 72 hai, the brown lesion became pronounced (Fig 2C). Also at early time points (12–48 hai) hyphae grew between live host cells, while after 72 hai the hyphal front grew between dying/dead host cells with a greater speed of advance (Fig 3B).

### *F*. *graminearum* transcriptome changes in the process of maize stalk infection

**Fig 4:**
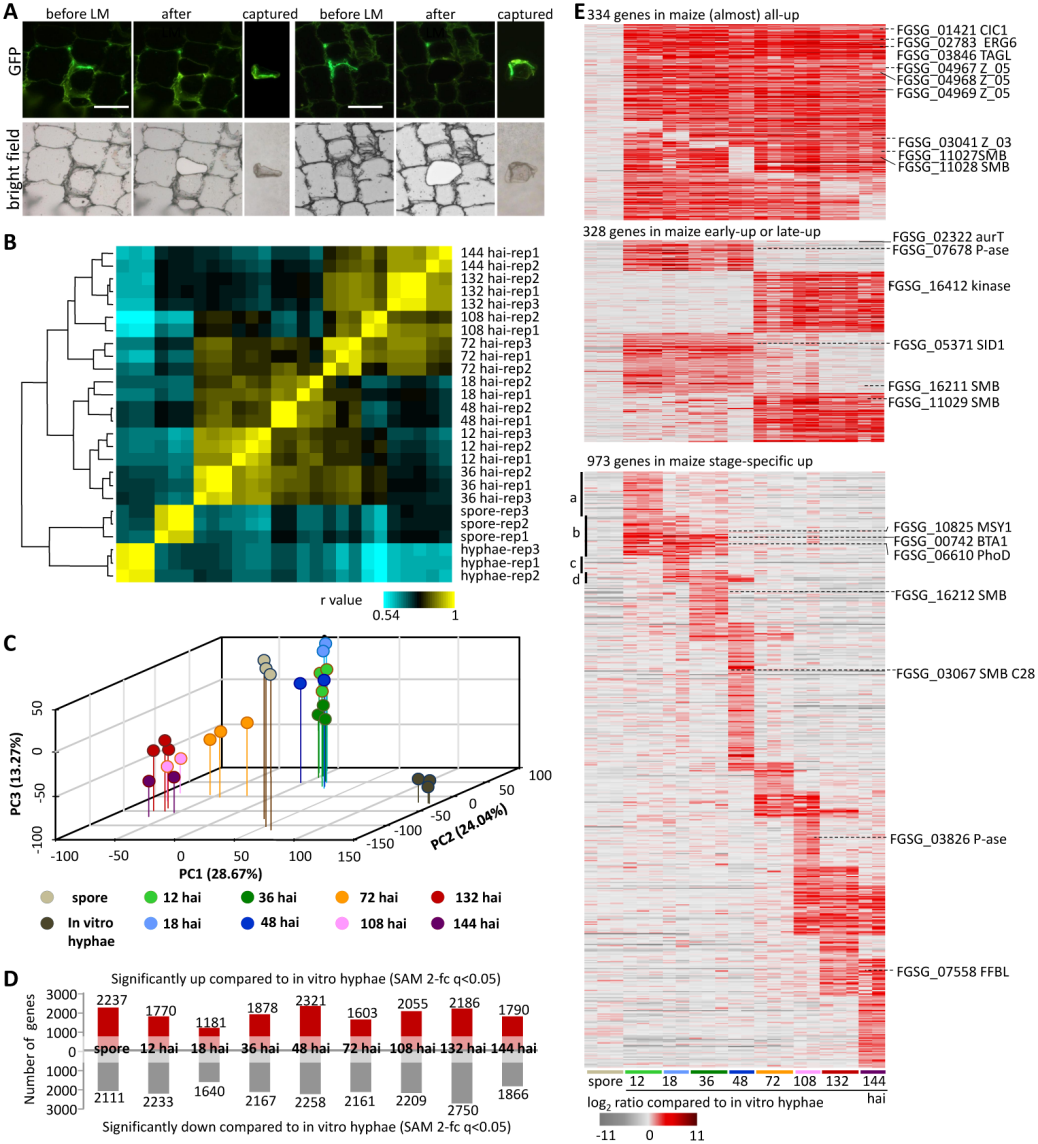
Laser microdissection-derived transcriptomes of *F*. *graminearum* in maize stalk. (A) Representative images of laser microdissection (LM) of *F*. *graminearum* from infected maize stalk pith at 72 hai (longitudinal sections). White bar = 100 μm. (B) A heat-map representation of the hierarchical clustering using Pearson correlation coefficients. (C) Principal components analysis using microarray data of all 13,346 genes. (D) Numbers of differentially expressed genes between hyphae in maize and hyphae *in vitro*. (E) Heat maps of genes that were significantly up-regulated in maize compared to *in vitro* hyphae; see Figure H in S1 Text for more.

To gain insight into how *F*. *graminearum* survives and proliferates inside the maize stalk, we charted the *in planta* fungal transcriptome dynamics along with disease development, using laser microdissection in combination with fungal microarray hybridization. Our previous experience studying cell behavior from cell type specific transcriptomes [39, 43] has reinforced that the predictive power of laser microdissection-derived transcriptome data relies on the homogeneity of the harvested cells. Maize stalks are composed of various cell types (Figure B in S1 Text), and *F*. *graminearum* progression in rind fiber cells or vascular bundles is different from the progression in pith parenchyma cells (Figure C in S1 Text). To tackle a complex process in a simple way, we focused on *F*. *graminearum* infection in parenchyma cells, which constitute the major cell type in the pith of maize stalk. We used laser microdissection to capture hyphal samples infecting maize stalk parenchyma cells at eight representative time points, (12, 18, 36, 48, 72, 108, 132, and 144 hai), carefully avoiding hyphae growing in rinds or vascular bundles (Fig 4A). RNA derived from these samples was hybridized to the *F*. *graminearum* whole genome Affymetrix GeneChip [44]. We thus obtained *in planta F*. *graminearum* transcriptome data at eight time points during maize stalk infection. *In vitro*-cultured spores used for inoculation represented the initial time point (0 hai), and hyphae cultured *in vitro* for 72 h represented the *in vitro* growth stage. RNA from these samples was also hybridized to the *F*. *graminearum* GeneChip to obtain *in vitro* fungal transcriptomes for comparison.

The much greater effort we invested in isolating fungal hyphae from infected stalks, rather than directly extracting RNA from bulk infected stalk, was rewarded by the quality of the data we obtained. At a very early infection stage, the fungal proportion of the infected tissue may be <0.1%, resulting in a very low signal-to-noise ratio in the transcriptomic data. In late infection stages after colonization, the fungal proportion could be much higher. A large range of fungal proportion between early and late infection stages makes it very difficult to chart true fungal expression changes over the course of the infection [45, 46]. However, by using laser microdissection, we were able to isolate enriched fungal samples throughout the infection process, and this allowed us to directly compare gene expression changes at each stage.

Unsupervised hierarchical clustering of *F*. *graminearum* transcriptomes showed that all the biological replicates at a given time point grouped together (Fig 4B) with high correlations (Dataset A in S2 Text), indicating the high reproducibility of our microarray data. We randomly chose 16 genes for real-time PCR verification at seven time points (spore, *in vitro* 72 h, 12 hai, 18 hai, 48 hai, 72 hai, and 132 hai), and results for 13 genes were highly consistent with the microarray data (Figure G in S1 Text).

Using the Significance Analysis of Microarrays (SAM) method [47], a false discovery rate of 0.05 was used as the cutoff value for statistical significance, and a 2-fold change in expression was used as the cutoff for fold changes. Within these bounds, we identified that at each *in planta* time point, an average of 1,848 genes were significantly up-regulated and an average of 2,160 genes were significantly down-regulated, compared to the reference *in vitro* growth stage (Fig 4D and Dataset B in S2 Text).

The expression data at eight time points were obtained from the most synchronous materials we could collect. Considering the total of 4,575 genes that were significantly up-regulated in at least one *in planta* time point, we found 394 groups that clustered based on their expression patterns over the course of infection from 0 hai to 144 hai (Dataset C in S2 Text). The heat maps in Fig 4E and Figure H in S1 Text show that 794 genes (17.4% of 4,575 genes) were up-regulated in at least seven out of the eight *in planta* time points, 1108 genes (24.2%) were up-regulated either in at least three out of the four early *in planta* time points (12 hai, 18 hai, 36 hai and 48 hai) or in at least 3 of the four later *in planta* time points (72 hai, 108 hai, 132 hai and 144 hai); and 1289 genes (28.2% of 4,575 genes) were up-regulated in only one *in planta* time point or in only two to three adjacent *in planta* time points, which indicates stage-specific expression features. The genes in the three major types mentioned above constitute 70% of the 4,575 genes. Among the 4,575 differentially-expressed genes (DEGs), 3,006 were not up-regulated in spores compared to *in vitro* grown hyphae (Fig 4E and Dataset D in S2 Text), representing the group of genes that most likely function in hyphal growth in a maize stalk. Thereafter, we term these 3006 *F*. *graminearum* DEGs up-regulated in at least one time point during maize stalk infection but not up-regulated in the spore sample (compared to *in vitro* hyphae) as genes preferentially expressed during hyphal growth in maize stalk (PEMS).

Based on Munich Information Center for Protein Sequences (MIPS) FGDB Functional Catalogue (FunCat) annotation [14, 48, 49], 5,147 *F*. *graminearum* genes are assigned to FunCat categories, 4,966 of the 13,346 genes presented on our microarray have assigned FunCat categories. A total of 163 FunCat categories are enriched in the 3,006 PEMS genes (detailed lists provided in Dataset E in S2 Text). Most of the enriched categories are in C-compound and carbohydrate metabolism (FunCat 1.05), amino acid metabolism (FunCat 1.01), lipid, fatty acid and isoprenoid metabolism (FunCat 1.06), secondary metabolism (FunCat 1.20), and extracellular metabolism (FunCat 1.25). Regarding subcellular localization of encoded proteins, extracellular/secreted protein (FunCat 70.27) are enriched at the 12–36 and 108–132 hai timepoints, while plasma membrane proteins (FunCat 70.02) are enriched in the 108–144 hai DEGs.

Considering the PEMS genes that are up-regulated in only one *in planta* time point, or in only two to three adjacent *in planta* time points (Fig 4E), we can observe over-represented functional categories defined as the FunCat classification system. For example, among genes significantly up-regulated at 12–18 hai (Fig 4E b), the enriched groups are metabolism of the cysteine—aromatic group (FunCat 01.01.09), phosphate metabolism (FunCat 01.04), C-compound and carbohydrate metabolism (FunCat 01.05), lipid, fatty acid and isoprenoid metabolism (FunCat 01.06), secondary metabolism (FunCat 01.20), cell cycle (FunCat 10.03), cellular transport (FunCat 20), transport routes (FunCat 20.09), cell rescue, defense and virulence (FunCat 32) (Dataset F in S2 Text).

### Expression patterns of genes for cell wall degradation are consistent with infection stage

**Fig 5:**
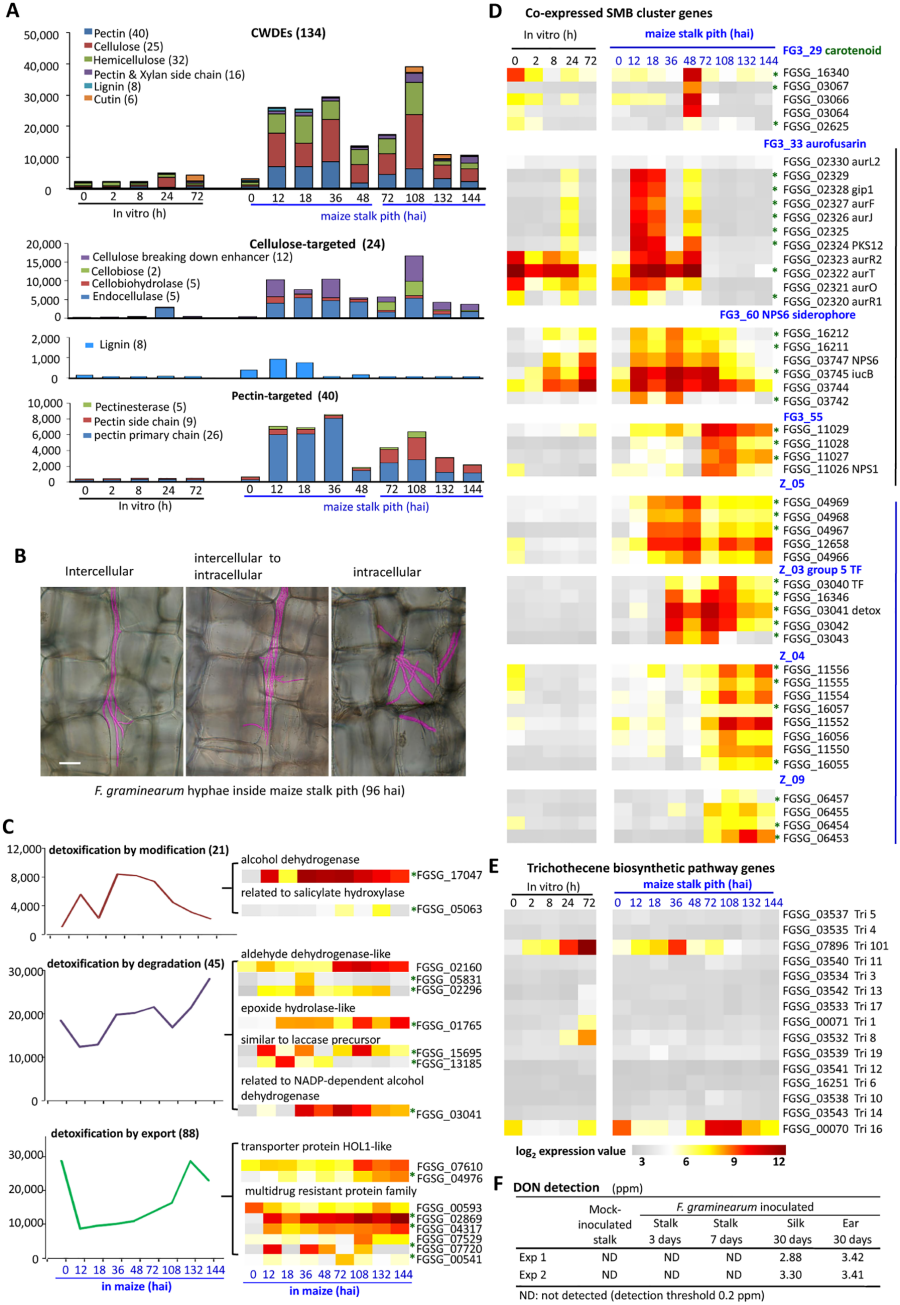
Time course of expression of genes potentially involved in plant cell wall degradation, detoxification, and secondary metabolite biosynthesis. (A) Expression of cell wall degradation-related genes grouped based on putative targeted components of plant cell wall. Total signal intensities for the genes in the denoted group are charted. (B) Hyphae in the pseudo pink color represent various growth path. (C) Expression patterns of detoxification-related genes grouped based on FunCat annotations. Total signal intensities for all genes in the denoted group are charted. The number of genes included in each category is indicated in parentheses. (D)-(E) Expression of several secondary metabolite biosynthesis clusters. hai: hours after inoculation. Genes significantly up-regulated in at least one time point but not up-regulated in spores are labeled with green asterisks. (F) DON detection in maize tissue extracts. ppm = mg/kg.

The enrichment of FunCat categories “C-compound and carbohydrate metabolism” and “extracellular metabolism” during maize infection implies the production of plant cell wall degrading enzymes. The *F*. *graminearum* genome encodes 134 putative carbohydrate-active enzymes [50] (CAZymes, www.cazy.org) with signal peptides that potentially degrade various plant cell wall components, including cellulose, hemicelluloses, pectin, lignin and cutin (Dataset G in S2 Text). As expected, the aggregate (i.e. total combined) expression of these 134 putative plant cell wall degradation enzymes (CWDEs) was more than tripled during infection compared with *in vitro* grown *F*. *graminearum* (Fig 5A). The expression of 98 of these CWDEs was significantly up-regulated in at least one time point in maize stalk infection. There were two peaks of elevated overall expression levels of these genes during maize stalk infection (shown in Fig 5A and detailed in Dataset B in S2 Text); the first peak was at 12 to 36 hai, the second peak was at 108 hai.

Considering the cell wall components potentially targeted during infection, the overall expression levels of genes encoding enzymes targeting lignin, for example, were low. As Figure B in S1 Text shows, lignin accumulation (as indicated by pink staining) could be observed in cell walls in vascular bundles, but not in cell walls of pith parenchyma cells. The low expression of lignin digestion enzyme genes [51] may result in low-level synthesis of the enzymes, and may explain why *F*. *graminearum* does not frequently break into vascular bundles. Pectin main chain degradation enzymes reached very high levels at the early time points of infection when *F*. *graminearum* is primarily elongating intercellularly (Fig 5B). This matches our expectations: pectin is the major component of the intercellular middle lamella and would therefore play a significant part in blocking intercellular advancement of *F*. *graminearum*, whereas endolytic pectinase activity of the fungus might allow hyphal passage.

In general, the expression patterns of CWDE genes suggest that the expression of pathogen partition enzymes is fine-tuned to allow the pathogen to adapt to the changing conditions of the maize stalk environment.

### Many fungal genes that are potentially involved in detoxification showed increased expression during maize stalk infection

FunCat category 32.07 detoxification, a branch of the main category FunCat 32 cell rescue, defense and virulence, was significantly enriched in PEMS genes (Dataset F in S2 Text). The expression of 167 out of the 391 *F*. *graminearum* genes annotated with the function “detoxification” was significantly increased during maize infection compared to that in *in vitro* grown hyphae. (Fig 5C and Dataset H in S2 Text). It is interesting to note that the aggregate expression of three subgroups of detoxification-related genes (i.e. genes encoding proteins related to detoxification by modification, degradation, and export) peaked at different time points (Fig 5C and Dataset H in S2 Text). Genes related to detoxification by modification (FunCat 32.07.03) including those encoding proteins potentially with oxidoreductase and/or isomerase activity showed the peak expression at 36 hai; expression of genes involved in detoxification by export (FunCat 32.07.05) including putative multidrug resistance proteins peaked at 132 hai, and genes involved in detoxification by degradation (FunCat 32.07.09) such as putative epoxide hydrolase, reached their maximum expression at 144 hai. These observations suggest that the fungus may have different priorities in using detoxification approaches in different stages of infection.

### Diverse secondary metabolite biosynthetic cluster genes are induced in maize

The FunCat category “secondary metabolism biosynthesis” (SMB) was enriched in PEMS genes. Two low molecular weight secondary metabolites have been identified as virulence factors of *F*. *graminearum*: the mycotoxin DON, a virulence factor in wheat [21]; and an extracellular siderophore, a conserved virulence factor in many pathogens whose role is to acquire nutrients or to protect from oxidative stress [52]. Notably, the extracellular siderophore triacetylfusarinine biosynthesis FG3_60 cluster genes, such as non-ribosomal peptide synthetase 6 (FGSG_03747) [53] and a putative siderophore permease *MIR1* (FGSG_03744) [54], were highly expressed from 12 to 108 hai during maize stalk infection (Fig 5D and Dataset I in S2 Text). The expression data accord with the hypothesis that *F*. *graminearum* secretes siderophores during maize stalk infection. DON biosynthesis genes (Fig 5E) were not induced during maize stalk infection up to 6 days after inoculation (dai). Furthermore, no significant DON accumulation was detected in 3 or 7 dai maize stalk tissues using an enzyme-linked immunosorbent assay (Fig 5F).

In addition, *F*. *graminearum* is capable of producing 10 other secondary metabolites, including terpenes, polyketides and nonribosomal peptides [55]. The expression of clusters responsible for producing carotenoid, malonichrome, ferricrocin, triacetylfusarinine, aurofusarin, orcinol and culmorin was detected in maize stalk infection (Dataset I in S2 Text). For example, the carotenoid (terpenoid pigment) biosynthesis gene cluster was induced at 48 hai. The aurofusarin (polyketide pigment) biosynthesis cluster was induced at 12 hai and maintained at a high expression level at 18 hai and 48 hai (Fig 5D). We also detected aurofusarin by mass spectrometry in infected maize stalk tissue. The expression of genes in clusters responsible for production of zearalenone, fusarielin or fusarin C was not significantly induced in maize stalk infection (Dataset I in S2 Text).

In fungi, most of the genes required for the biosynthesis of a particular secondary metabolite occur in a cluster [56]. A total of 67 SMB clusters, including 10 with known products and others with unknown products, have been previously reported in *F*. *graminearum* [55,57,58]. Thirty-six out of these clusters showed a co-expression pattern in maize stalk infection in our data. We also found 7 additional coexpressed gene clusters based on expression profiles of adjacent genes (Dataset I in S2 Text).

### Expression of genes encoding enzymes potentially producing non-phosphorous lipids are induced at early infection time points

We observed that the aggregate (i.e. combined total) expression of 36 genes whose function is assigned to be in the metabolism of glycerophospholipids, essential components of all biological membranes, was actually lower during maize infection than in *in vitro* grown hyphae (Dataset J in S2 Text). But membrane biogenesis is essential for fungal hyphal growth in all conditions, and transcripts significantly up-regulated at early time points in maize stalk infection included several annotated as being involved in lipid, fatty acid and isoprenoid metabolism (Dataset F in S2 Text). So we explored whether this could be associated with active biosynthesis of membrane lipids other than glycerophospholipids.

**Fig 6:**
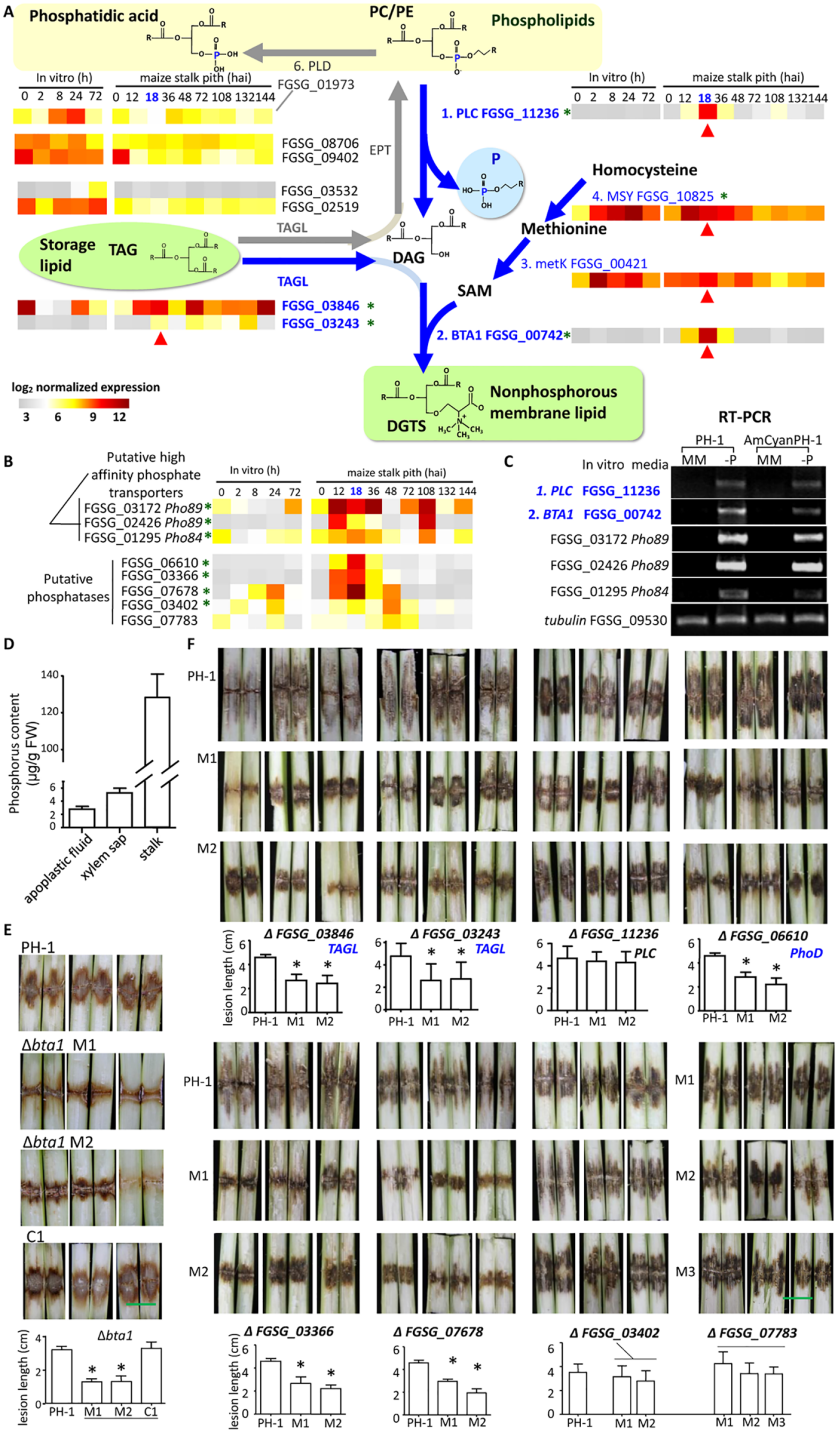
Expressional and functional analysis of genes potentially related to non-phosphorus membrane lipid biosynthesis and phosphate metabolism. (A) Diagram illustrating reactions related to the interconversion of lipids, and the expression of related genes. Heat maps indicate the expression of individual genes from microarray data. h: hour. hai, hours after inoculation. (B) Heat maps of genes related to phosphate transport and metabolism. Red arrows point to 18 hai, green asterisks indicate gene expression significantly up-regulated in maize infection compared to *in vitro* growth (SAM FDR < 0.05). (C) RT-PCR analysis of *in vitro* grown *F*. *graminearum*. MM: minimal medium, −P: minimal medium lacking phosphate. (D) ICP-MS measurements of phosphorus contents in various fractions of maize stalk. (E)-(F) Maize stalk infection assays. Lesions were diagnosed at 7 days post inoculation. *Significantly different difference from wild type-caused lesion (Student’s *t*-test, P <0.05), n = 3 independent experiments. Error bars denote SE. PC: phosphatidylcholine, PE: phosphatidylethanolamine, TAGL: triacylglycerol lipase, DAG: diacylglycerol, SAM: S-adenosylmethionine, DGTS: betaine lipid diacylglyceryl-N,N,N-trimethylhomoserine, PLC: phospholipase C, PLD: phospholipase D, MetK: methionine adenosyltransferase ETH-1, MSY: methionine synthase, EPT: ethanolamine-phosphotransferase, BTA1: betaine lipid biosynthetic enzyme.

Fig 6A shows the expression of genes encoding enzymes responsible for reactions involving diacylglycerol (DAG), which is a major precursor for the glycerophospholipids phosphatidylcholine (PC) and phosphatidylethanolamine (PE). FGSG_09402 and FGSG_08706, encode putative ethanolamine-phosphotransferases responsible for converting DAG to PC and PE. Both genes were expressed at lower level during maize infection than during germination *in vitro*. Particularly, the expression of FGSG_09402 at 12–18 hai, was <30% of the level in *in vitro* hyphae (Dataset J in S2 Text). The expression of FGSG_11236, which encodes a putative non-specific type phospholipase C (PLC) responsible for converting PC to DAG, was significantly increased at 12–18 hai, and increased more than 80-fold at 18 hai compared with its expression in *in vitro* hyphae. Triacylglycerol lipases (TAGL) are responsible for mobilizing triacylglycerol (TAG), the major storage lipid in fungi, to DAG. *F*. *graminearum* genome contains four genes encoding putative TAGLs. Two of these (FGSG_03846 and FGSG_03243) were significantly up-regulated in maize infection compared with *in vitro*. The above gene expression suggests an increase in DAG production and a reduction in DAG consumption, at 12–18 hai in maize infection.

This prompted us to consider what is the next intermediate after DAG in membrane lipid synthesis by *F*. *graminearum* in maize stalk if it is not PC or PE. We noted differential expression of FGSG_00742 encoding a putative S-adenosylmethionine:diacylglycerol 3-amino-3-carboxypropyl transferase (also called betaine lipid synthase, BTA1). This enzyme is responsible for the conversion of DAG to diacylglyceryl-N,N,N-trimethylhomoserine (DGTS). DGTS is a phosphorus-free lipid, but resembles the glycerophospholipid PC in structure and physical phase behavior [59]. The expression of FGSG_00742 at 18 hai was very significantly higher than that in *in vitro* growth, and more than 200-fold higher than in spores (0 hai). Almost no expression of *BTA1* was detected during *in vitro* germination and hyphal growth (at 0, 2, 8, 24, or 72 h). Real-time PCR experiments confirmed that expression of *BTA1* and *PLC* was much higher in maize 18 hai than *in vitro*-grown hyphae (Figure I in S1 Text).

In addition to DAG, BTA1 uses S-adenosylmethionine (SAM) as a cosubstrate in the production of DGTS. The gene encoding the enzyme responsible for SAM production from methionine is predicted to be FGSG_00421, which was expressed at high levels in most samples examined and exhibited a 1.7-fold increase (at 18 hai) over the level in spores (Fig 6A). The expression of the methionine synthase MSY1 gene (FGSG_10825) also increased approximately eightfold at 12 hai, and maintained a fourfold increase at 18 hai over the level in spores. Thus, the enzymes supplying the BTA1 substrate SAM had an elevated expression level at 18 hai, the point at which *BTA1* expression reached its peak.

We also checked the expression of genes encoding enzymes involved in glycerophospholipid metabolism that carry out reactions without net consumption or release of phosphorus. The expression of the gene encoding phospholipase D (FGSG_01973), an enzyme that digests membrane lipids but does not release phosphate groups, was lower at 12–18 hai than in spores and *in vitro* grown hyphae (Fig 6A). Along with the observation that the expression of *PLC* and *BTA1*, which encode enzymes converting phospholipid to phosphorus-free lipid, was higher at 18 hai than in spores and *in vitro* grown hyphae, these results hinted at a reduction in phosphorus content in membrane lipids composition at 18 hai in maize stalk infection.

The FunCat category “phosphate metabolism” is enriched in DEGs up-regulated in maize 12–18 hai but not up-regulated in the spore sample (compared to *in vitro* grown hyphae) (Dataset F in S2 Text). Several genes encoding putative phosphatases are preferentially expressed during hyphal growth in maize stalk (Fig 6B). For example, FGSG_06610 encodes a putative protein with a conserved phosphodiesterase/alkaline phosphatase D (PhoD) domain, and high similarity to PhoD from the cyanobacterium *Aphanothece halophytica* (overall 35% identity and 50% similarity; Figure J in S1 Text), an enzyme that is responsible for the release of free phosphate from organic compounds [60]; FGSG_03366 encodes a putative secreted protein with a conserved histidine phosphatase domain (branch 2) found in histidine acid phosphatases and phytases and a His residue that is phosphorylated during the reaction (similar to Pho12 in budding yeast); FGSG_07678, encodes a secretory acid phosphatase domain belonging to the aspartate-based protein phosphatase family.

### Expression of genes encoding putative phosphate transporters increased during maize stalk infection

Our expression analyses suggested a hypothesis in which *F*. *graminearum* conserves phosphorus by producing phosphorus-free membrane lipids so as to use as little phosphorus as possible but still maintain hyphal growth during the early time points of maize stalk infection. We further propose these changes are a response to phosphorus starvation.

Phosphorus starvation responses have been studied extensively in the fungus *Saccharomyces cerevisiae* [61–63], and it was observed that the plasma membrane high-affinity phosphate importers PHO89 and PHO84 are induced at the transcriptional level in phosphorus-limited conditions [61, 62], and that PHO84 plays a role in sensing phosphate [63]. The PHO89 homologous genes in *F*. *graminearum*, FGSG_03172 and FGSG_02426, were highly induced at 12 hai (before 18 hai when *BTA1* and *PLC* reached peak expression levels), while the expression of the gene homologous to PHO84 (FGSG_01295) increased by 12 hai and continued to increase, peaking at 18 hai (Fig 6B). In addition, RT-PCR analysis showed that expression of the genes encoding these phosphate transporters was induced when *F*. *graminearum* was grown in phosphorus-depleted medium, as was expression of *BTA1* and *PLC* (Fig 6C). These results are consistent with the idea that *F*. *graminearum* is confronting phosphate limited conditions 12–18 hai in maize stalk, at a time when it is mostly growing intercellularly in pith tissues surrounded by living maize cells.

### Maize stalk apoplast is phosphorus limited

We then used inductively coupled plasma mass spectrometry (ICP-MS) to measure the phosphorus content in maize stalk, which was approximately 120 micrograms per gram fresh weight, equivalent to about 3 mM phosphorus (Fig 6D), which is in the same range as in routine minimal medium (7 mM). However, because phosphorus is vital to plant growth, living plants tightly preserve their cellular phosphorus content, and the apoplastic concentration of phosphorus is thought to be low [64]. So we analyzed stalk apoplastic fluid of a mature maize plant, by first collecting the xylem sap from excised stalk tissue using hydraulic pressure, then collecting apoplastic fluid by vacuum infiltration and centrifugation. The residual stalk tissues were ground into powder to measure non-apoplastic phosphorus (Figure K in S1 Text). Using ICP-MS, the measured phosphorus content in apoplastic fluid was below 3 micrograms per gram fresh weight, i.e., much lower than the stalk tissue phosphorus content (Fig 6D and Figure L in S1 Text). Furthermore, the measured volume of a 6-gram stalk tissue sample was about 8 cm^3^, while the apoplastic space was about 20% of the total stalk volume, meaning that the phosphorus concentration in the stalk apoplastic space was 0.3 mM, about one-tenth of the intracellular concentration (Fig 6D). This demonstrates that the phosphorus content is indeed low in the apoplastic space of maize stalk and intercellularly invading *F*. *graminearum* is faced with a phosphorus shortage.

### Deletion of putative phosphatase genes, TAG lipase genes or BTA1 reduced *F*. *graminearum* virulence in maize stalk infection

To further evaluate the involvement of the putative non-phosphorus membrane lipid production enzymes and phosphatases in *F*. *graminearum* infection of maize stalk, we generated deletion mutants for *BTA1*, TAG lipases FGSG_03846 and FGSG_03243, *PLC*, and phosphatases FGSG_06610, FGSG_03366, FGSG_07678, FGSG_03402, and FGSG_07783 (Figure M in S1 Text). All nine gene deletion mutants grew similarly to the wild-type strain on routine medium, but six showed significant reductions in the size of lesions at 7 days after inoculation into maize stalk (Fig 6E and 6F). Multiple independent mutants with FGSG_03846 or FGSG_03243 putative TAG lipase gene knockouts produced a 40% reduction in lesion size relative to the wild type, while deletion of *PLC* had no effect on lesion size. Lesions caused by mutants in FGSG_06610, FGSG_03366 or FGSG_07678 were about 50% as large as wild-type lesions, but deletion of the other two putative phosphatase genes, FGSG_03402 and FGSG_07783, did not significantly affect maize stalk infection.

**Fig 7:**
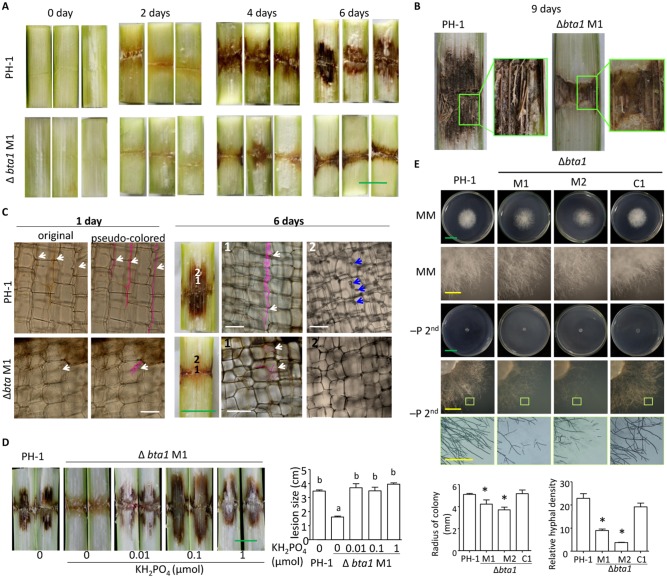
*graminearum* bta1 mutants show reduced virulence in maize stalk infection and reduced growth on phosphate depleted media. ***F***. (A)-(C) Maize stalks infected by wild-type or mutant *F*. *graminearum* at the indicated number of days after inoculation. Note that more vascular strands are visible in the stalk infected by wild-type *F*. *graminearum* than that infected by mutant *F*. *graminearum* in (B). White arrows point to hyphae (in pseudo pink). Blue arrows point to debris-like structure in host cells. (D) Supplementation with KH_2_PO_4_ increased lesion size caused by the bta1 mutant strain. Different letters indicate statistical differences among the strains, according to Tukey’s multiple comparison test (P = 0.05), n = 3 independent experiments. (E) Fungal growth after 2 days of culture on various media. MM: minimal medium, −P: minimal medium lacking phosphate. −P^2nd:^ hyphal disks from −P medium were transferred to fresh −P medium plates to exhaust endogenous phosphate. Statistical analysis for colony radius and relative hyphal densities are charted at the bottom of the panel. The radius was measured at 1 day after transfer onto the −P^2nd^ medium. The relative hyphal density was determined using ImageJ software by measuring the gray value of the same positions of each fungal colony on pictures (subtracted from the respective background gray value). *Significantly different from wild type (P < 0.05) according to Student’s *t* test, n ≥3. Error bars denote SE.

Of these nine genes, the deletion of BTA1 affected maize stalk infection most severely. When examined 7 days after inoculation, the lesions caused by two independent *bta1* mutant strains were reduced to approximately one-third the size of lesions caused by the wild-type strain; complemented strains produced lesions that were similar in size to those caused by the wild-type strain (Fig 6E). When infected maize stalks were left open to air to dry for two more days (i.e. until 9 dai), it was apparent that fewer vascular bundles were exposed in lesions caused by the *bta1* mutant than in lesions caused by the wild-type fungus (Fig 7B). Therefore, BTA1 is important for the virulence of *F*. *graminearum* in maize stalk infection.

### *BTA1* deleted *F*. *graminearum* did not grow intercellularly in maize stalk

To understand how the deletion of *BTA1* attenuates *F*. *graminearum* colonization of maize stalk, we compared the stalk infection progress of mutant and wild-type fungus at 2, 4 and 6 dai. Fig 7A shows that the mutant and wild-type exhibited differences as early as 2 dai; stalk inoculated with the wild-type strain had already turned brownish at the infection site, whereas stalk tissues inoculated with mutant strains were still of similar color to mock-inoculated stalks. This makes sense as *F*. *graminearum BTA1* expression is induced as early as 18 hai in infection of maize stalks by the wild-type (Fig 6A). By 4 dai, the stalk tissues infected by the mutant strain were brown, but the brown region was limited to 3 mm above and below the inoculation site on both up and down sides, while the brown region caused by wild-type strain usually reached 6 mm. By 6 dai, the differences in brown lesion sizes were more evident, and the debris-like structures observed in maize cells surrounding wild-type hyphae were not seen in maize cells surrounding *bta1* mutant hyphae (Fig 7C). In addition, hyphae were generally observed in the intracellular regions of lesions caused by *bta1* mutants, while in lesions caused by the wild type, the hyphal front migrated intercellularly (Fig 7C). When we examined the fungal progression as early as 1 dai, we found that the mutant hyphae were growing intracellularly, while most wild-type hyphae were growing intercellularly. This result indicates that the capacity for intercellular expansion is compromised in the *bta1* mutant.

### Phosphate supplementation restored the virulence of the *bta1* mutant

If our hypothesis is correct that the *bta1* mutant’s inability to cope with phosphorus-limitation compromises its virulence in maize stalks, then supplementation of phosphate during the early phase of invasion should restore its virulence. Phosphate supplementation applied via soil mix did not affect lesion size for either mutant or wild-type strains, but it may be difficult to affect apoplastic phosphate levels in this way. However, when we added a KH_2_PO_4_ solution to the infection site 8 h after inoculation of *bta1* mutant spores, the lesion size observed at 7 dai significantly increased, and was similar to those inoculated by wild-type *F*. *graminearum* (Fig 7D and Figure N in S1 Text). The results showed that, as little as 10 nmol exogenous phosphate restored the virulence of the *bta1* mutant in maize stalk.

### Knockout of the BTA1 gene impairs *F*. *graminearum* growth under extreme phosphate starvation *in vitro*

We next assessed the viability of the *BTA1* knockout strains on phosphorus depleted medium. *bta1* mutant colonies grew on minimal medium and had similar expansion rates to the wild type (Fig 7E), although they accumulated red pigmentation earlier than the wild type. Both the mutant and wild-type strains grew slowly on phosphate depleted medium than on complete medium. We suspected that phosphorus stored in the original spore persisted and enabled growth. Therefore, we transferred hyphae from phosphate-deprivation medium to fresh phosphate-deprivation medium for a second round of growth. Fig 7E shows that in this second round growth, the *bta1* mutants barely grew hyphae, while the wild-type and complemented strains produced some new hyphae. These results demonstrate that without BTA1, the growth of *F*. *graminearum* was impaired under extreme phosphate starvation *in vitro*.

### *F*. *graminearum* BTA1 can produce the phosphorus-free membrane lipid DGTS

**Fig 8:**
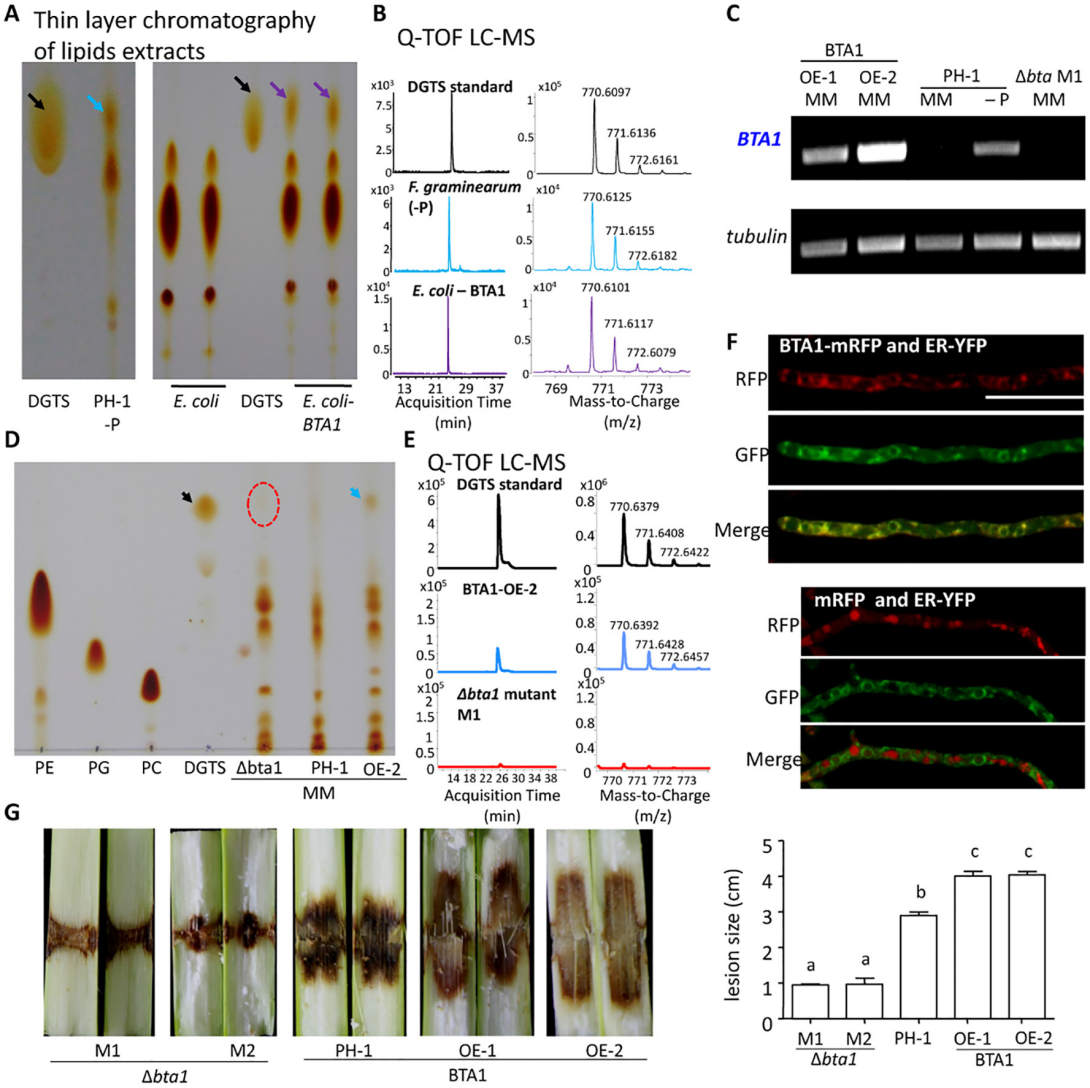
BTA1 is responsible for DGTS production. (A), (D) Thin layer chromatography (TLC) of lipid extracts from *F*. *graminearum* or *E*. *coli* expressing *F*. *graminearum* BTA1. (B), (E) Spots scraped from TLC plates (indicated by arrows) were applied to accurate-mass quantitative time-of-flight liquid chromatography mass spectrometry (Q-TOF LC MS) analysis. See Figure I in S1 Text for more lipid analyses. (C) RT-PCR results for *BTA1* expression in various *F*. *graminearum* strains and conditions. (F) Subcellular localization of BTA1-mRFP in *F*. *graminearum*. ER: endoplasmic reticulum localized protein marker. White bar = 100 μm. (G) Virulence assays on maize stalks. Lesions were measured at 7 days after inoculation. Different letters indicate statistical differences among the strains according to Tukey’s multiple comparison test (P = 0.05), n = 3 independent experiments.

The putative BTA1 in *F*. *graminearum* is similar to BTA1 in *Neurospora crassa* (overall identity 36%, Figure I in S1 Text). *N*. *crassa* BTA1 is responsible for synthesis of the phosphorus-free betaine lipid DGTS upon phosphate starvation [59]. *BTA1* gene expression in *F*. *graminearum* is induced in response to phosphate starvation *in vitro* (Fig 6C). We directly assessed whether *F*. *graminearum* BTA1 can, like its homolog in *N*. *crassa*, synthesize DGTS. The lipids extracted from *F*. *graminearum* hyphae grown on phosphate-deprived medium for 14 days were analyzed by thin layer chromatography (TLC). Fig 8A and 8B shows that a spot migrating at a similar position to DGTS was detected from wild-type hyphae; this was confirmed to be DGTS by quadrupole time-of-flight liquid chromatography–mass spectrometry (Q-TOF LC-MS). DGTS could not be detected in the extracts from *bta1* mutants grown on either phosphate-replete or phosphate depleted medium (Fig 8D and Figure I in S1 Text).

*BTA1* cDNA obtained from *F*. *graminearum* was fused with GST and expressed in *Escherichia coli*. DGTS production in this system was similarly detected by TLC and Q-TOF LC-MS (Fig 8A and 8B and Figure I in S1 Text). Furthermore, we constructed a *F*. *graminearum* strain constitutively expressing BTA1 (BTA1 OE; Fig 8C). DGTS could be detected in extracts from this strain grown on minimal medium (Fig 8D and 8E). These results demonstrate that *F*. *graminearum* BTA1 is responsible for DGTS biosynthesis.

We investigated the subcellular localization of *F*. *graminearum* BTA1 (Fig 8F) and detected it in the endoplasmic reticulum (ER) where it colocalized with an ER marker protein fused to YFP [65]. The BTA1 product of *Chlamydomonas reinhardtii* is also thought to localize to the ER [66], and the ER is a major location for membrane lipid biosynthesis.

BTA1 OE strains express *BTA1* at the spore and germinated spore stages, i.e. earlier than in the wild type, which induces BTA1 expression at 18 hai. BTA1 OE strains caused even bigger lesions than the wild-type strain in maize stalks (Fig 8G and Figure N in S1 Text), suggesting that the ability to conserve phosphate through BTA1 activity at an earlier stage of growth can actually enhance *F*. *graminearum* virulence in the phosphorus-limited microenvironment of maize stalks.

## Discussion

**Fig 9:**
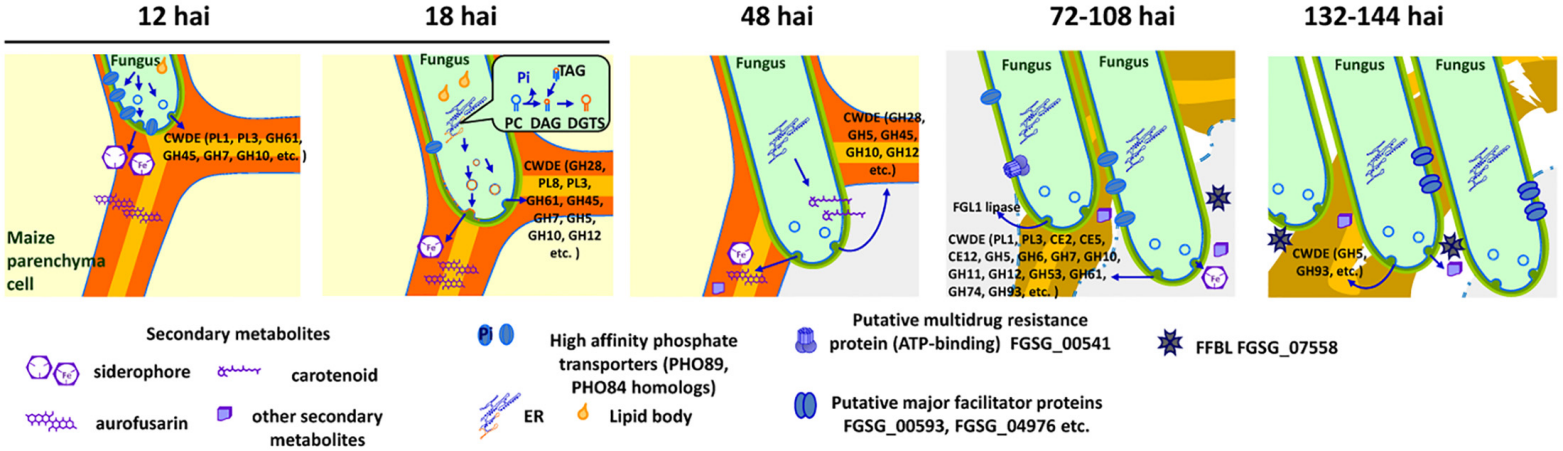
A hypothetical model of molecular events during maize stalk infection by *F*. *graminearum*, based on microscopic observation and gene expression profiling. At 12 h after inoculation (hai), the fungus grows between live parenchyma cells, secretes plant cell wall degrading enzymes (CWDE) mainly breaking primary chains of pectin (PL1, PL3), cellulose (GH61, GH45, GH7) and hemicellulose (GH12) for penetrating barriers, secretes secondary metabolites (siderophore and aurofusarin), and increases high affinity phosphate transporters; at 18 hai, the fungus continues to grow between live parenchyma cells, secretes similar CWDEs and secondary metabolites, but reduces the expression of high-affinity phosphate transporters, and deploys BTA1 to produce non-phosphorus membrane lipids to assure fast growth under phosphate starvation; at 48 hai, the fungus starts to grow into host cells and kills invaded and surrounding host cells, gains access to host cellular phosphate, reduces CWDE production as intracellular growth needs less cell wall breakage, and produces secondary metabolites such as carotenoids; around 72–108 hai, more CWDEs (including those targeting side branches of pectin) and FGL1 lipase are produced for full digestion of plant tissues, a toxic lectin FFBL is secreted, and a putative multidrug resistance protein is also produced; around 132–144 hai, the fungus grows among dead parenchyma cells, produces less CWDEs, more FFBL, and more major facilitator proteins. CAZy categories are indicated in parentheses after the respective CWDEs.

In this study, we performed in-depth anatomical studies and laser microdissection-assisted fungal transcriptomic profiling on the development of an important disease, maize stalk rot caused by *F*. *graminearum* (Figs 1–4). These data provided many hypotheses regarding the mechanisms used by *F*. *graminearum* to confront host environments, including cell wall degradation, detoxification, and the generation of diverse secondary metabolites (Fig 9). Most excitingly, following up on our observation of changes in the expression of membrane lipid metabolism pathways, we showed experimentally that *F*. *graminearum* uses betaine lipid synthase (BTA1) to produce non-phosphorus DGTS (instead of PC) for use in membrane lipids during early time points of maize stalk infection, and we have correlated this change with enhanced ability to overcome phosphate limitation in the apoplast of maize stalk tissue (Figs 6–8). Therefore the non-phosphorus membrane lipid DGTS and the enzyme BTA1 might be potential targets for future design of *F*. *graminearum* resistance in maize.

Our results reveal a previously unreported stress for apoplastic pathogens inside healthy maize stalks, i.e., phosphate limitation. Even when the whole maize plant is not phosphate-limited, the apoplastic phosphorus level is low (Fig 6D). Pathogens that take an extracellular route in early infection have to confront this stress. In our wounding inoculation system, *F*. *graminearum* spores started to germinate around 8 hai. *In planta* profiling hinted that, shortly after germination (at 12 hai), intercellular *F*. *graminearum* may, in a common phosphate starvation response, produce three putative high-affinity phosphate transporters (Fig 6B) to fetch environmental phosphate. However, as most host cells have intact plasma membranes (based on our plasmolysis results—Fig 3B) and the host extracellular space is phosphorus limited (Fig 6D), the fungus still may not obtain enough phosphate by the use of transporters to support rapid hyphal growth. We propose that a more specialized strategy is used by *F*. *graminearum* around 18 hai, allowing more sparing use of phosphorus-containing PC to support a vigorous intercellular advance. Without this capability (as suggested by the BTA1 knockout mutant), *F*. *graminearum* may still make progress by intracellular growth, but with only very slow fungal expansion (Fig 7C). Between 36 and 48 h after infection, we observed a reduction in *BTA1* expression, which is consistent with the onset of intracellular growth and the concomitant liberation of phosphate from host cells that have lost membrane integrity. Later on, although hyphae at the front still travel intercellularly, the surrounding cells have already lost plasma membrane integrity (Fig 3B) and it is possible that cellular phosphate leaks into the intercellular space and thus is available to the fungus.

Using BTA1 to survive phosphate starvation has also been reported in non-pathogens, such as *N*. *crassa* [59]. It has been reported that DGTS accumulation is under the control of the transcriptional activator NUC-1. During maize infection, FGSG_01438, a homolog of NUC-1, is also expressed in *F*. *graminearum*, although its expression does not increase. As Figure O in S1 Text shows, the bta1 promoter (1 kb immediately upstream of the start codon) contains several sequences that are very similar to phosphate starvation response elements conserved among fungi [67].

Our results highlight the importance of adaptation to a challenging microenvironment during the early phases of host infection. Many fungal pathogens, including several maize stalk rot pathogens, possess the *BTA1* gene (Figure P in S1 Text). Maize is known for locking up phosphate in the form of phytate (also called inositol hexakisphosphate). Phosphorus starvation might be a common hurdle to maize stalk fungal pathogens. The maize anthracnose stalk rot fungus *Colletotrichum graminicola* increased expression of genes encoding putative secreted phytases when it infected maize stalk [45, 68], suggesting *C*. *graminicola* may gain access to phosphate from phytate.

Our results lead to the prediction that the membrane of fungal hyphae around 18 hai in maize stalk contains more DGTS than hyphae grown *in vitro*. Although DGTS resembles PC in many ways, it is not cleaved by lipase PLC or PLD, while PC can be cleaved by PLC and PLD to release fatty acids, which might be used as signaling molecules in communication between the plant host and the fungal pathogen [69]. Given that fatty acid-derived signals play roles in plant defense [70], the changes of fungal membrane lipids possibly have effects on plant defense. These are speculations requiring further exploration.

*F*. *graminearum* also causes wheat head blight disease. Among the pathogenic genes reported in wheat head blight infection, only the cyclin-dependent kinase CDC2A has been reported to also function in maize stalk infection [33]. Among other pathogenic genes reported in wheat head blight infection, we found that the expression of FGL1 [32], a putative protein kinase Fg04770 [34], Rab2 [35], three putative phosphatases [36] and the putative transcription factor FGSG_01915 [37] was significantly increased expression during maize stalk infection compared to *in vitro* growth (Dataset K in S2 Text). This suggests these genes might also participate in maize stalk rot infection.

The mycotoxin DON is required for wheat head blight infection [21], but DON biosynthesis genes except *Tri101* and *Tri16* were not induced during maize stalk infection up to 144 hai (Fig 5E). Trichothecene 3-O-acetyltransferase (Tri101) catalyzes the conversion of toxic trichothecenes to less-toxic products; and has been proposed as a metabolic self-protection mechanism in *F*. *graminearum* [71]. Furthermore, the metabolite DON could not be detected in infected maize stalks up to 7 days post inoculation. Our results suggest the role of DON in early infection of maize stalk might not be as important as it is in wheat head infection.

Several reports describe *F*. *graminearum* transcriptomes during head blight development in cereals using RNA extracted from bulk infected plant tissues and the same design of GeneChip as in this study [44,72,73]. Lysoe and colleagues studied *F*. *graminearum* gene expression at 24, 48, 72, 96, 144 and 192 hai during wheat head infection [72], and compared with *F*. *graminearum* gene expression at 24, 48, 72, 96 and 144 hai during barley head infection, as well as during *in vitro* growth on three types of media [44]. Because of sampling method differences, direct global normalization of transcriptomes from our work and those from wheat or barley head blight infection may not deliver meaningful conclusions. However, Lysoe and colleagues generated three lists of *F*. *graminearum* genes based on their expression: 1. 404 probe sets (corresponding to 355 genes) named “exclusively” in wheat, are those whose expression can be detected in wheat head infection but not detected in barley head infection or during growth in media; 2. 113 probe sets named “exclusively” in barley, are those whose expression can be detected in barely head infection but not in wheat or in media; 3. 395 probe sets (corresponding to 369 genes) named “both in wheat and barley” whose expression was detected both in wheat and barley head infection but not in media. Comparing these gene lists derived from wheat and barley head blight infection to our list of genes with significantly increased expression during maize stalk infection than *in vitro* grown hyphae may provide insights into common and different pathogenesis strategies in wheat/barely head blight and maize stalk rot above the level of individual genes. For example, among the 404 and 113 probe sets detected exclusively in wheat and barley, respectively [72], 82 and nine, respectively, were also up-regulated in at least one time point in maize stalk infection; while in the 369 genes expressed in both wheat and barley, 323 were also significantly up-regulated in at least one time point in maize stalk infection (Figure Q in S1 Text and Dataset L in S2 Text). These 323 common genes could be candidate core pathogenesis genes for *F*. *graminearum* infection in various hosts. Dataset K in S2 Text also provides detail on the expression in maize stalk of previously reported SMB related genes [72].

Lysoe et al. [72] further reported that the 355 *“*exclusively” expressed in wheat genes are enriched in 18 FunCat groups including allantoin and allantoate transport, degradation of polysaccharides and ester compounds, and so on. We identified 163 enriched FunCat groups in 3006 genes that were significantly up-regulated in maize stalk infection but not up-regulated in spores (relative to *in vitro* grown hyphae). Dataset M in S2 Text provides detailed comparisons between [72] and our work. Two-thirds of the 18 functional groups enriched in wheat infection are also enriched in maize stalk infection. For example, the functional group predicted to be involved in FunCat 20.01.23 allantoin and allantoate transport is also enriched during maize infection with genes significantly up-regulated at 108, 132 and 144 hai. For another example, FunCat 01.25 extracellular metabolism has been identified as enriched in both wheat head infection and maize stalk infection, our study pointed out that more branches of FunCat 01.25 extracellular metabolism are enriched at 12–18 hai in maize stalk infection, including 01.25.07 exogenous polysaccharide degradation, extracellular polysaccharide degradation, 01.25.09 extracellular lignin degradation (Dataset M in S2 Text).

Lysoe et al. [72] also reported that in the 395 probe sets expressed in both wheat and barley, the over-represented functional categories were carbohydrate metabolism, extracellular ester and polysaccharide degradation, polysaccharide binding, disease, virulence and defense and secondary metabolism. These functional categories are also enriched in genes up-regulated during maize stalk infection.

A Fungal Fruit Body Lectin (FFBL, FGSG_07558) protein has been identified as a fungal effector that causes plant cell death in Arabidopsis [74]. We found its expression increased around the late colonization phase of maize stalk infection by *F*. *graminearum* (after 108 hai; Dataset K in S2 Text), suggesting this protein might also function in late-stage maize stalk infection. The biotrophic basidiomycete pathogen *Ustilago maydis* uses both common core effectors and organ-specific effectors to colonize its host [75, 76]. We previously used a similar approach to that in this work to profile *F*. *graminearum* expression in a stage-specific manner during infection of coleoptiles of wheat seedlings at 16, 40 and 64 hai [38]. Global comparison of dynamic transcriptomes provides a chance to comprehensively illustrate the overlapping but distinct infection strategies of *F*. *graminearum* during the infection of the coleoptiles of wheat seedlings and the pith tissues of mature maize stems. Comparisons suggest that, in wheat coleoptiles and maize stalks, *F*. *graminearum* may use similar cell surface molecules such as eight-cysteine-containing fungal extracellular membrane (CFEM) proteins, putative fungal cell surface proteins containing a [SG]-P-C-[KR]-P motif, glycosylphosphatidylinositol-anchored proteins and G protein-coupled receptors (GPCRs), as well as secreted peptidases (Figure R, S in S1 Text, Dataset N in S2 Text). This is consistent with the result that *F*. *graminearum* strains with *CFEM1* deleted exhibited reduced virulence in both wheat coleoptiles [39] and maize stalk infections, while a complemented stain resumed full virulence (Figure W in S1 Text). However, the use of secondary metabolite biosynthesis clusters, detoxification genes and membrane lipid remodeling enzyme genes was different between infection of maize stalk and wheat coleoptile (Figure T, U, V in S1 Text), with essentially more diverse and higher expression in maize stalk infection than in wheat coleoptile infection. This indicates that *F*. *graminearum* might produce more secondary metabolites (and produce them earlier) during maize stalk infection than during wheat coleoptile infection.

Furthermore, we also examined the virulence of our *bta1* mutants during wheat coleoptile infection and found no significant difference in lesion size between the mutant and wild-type fungus (Figure X in S1 Text), consistent with the result that *BTA1* expression was lower during wheat coleoptile infection than during maize stalk infection (Dataset J in S2 Text). Figure L in S1 Text shows that, as in maize stalk apoplast, the phosphorus content in wheat coleoptiles apoplast is low. It is possible that *F*. *graminearum* in wheat progresses more quickly to intracellular growth than in maize or that it utilizes other strategies to conserved phosphorus that do not involve BTA1.

## Methods

### Maize stalk infection and microscopic observation

The *F*. *graminearum* strain AmCyanPH-1 [41] was previously generated by our lab from the sequenced strain PH-1 (NRRL 31084, [13]), and is isogenic to PH-1 in all respects except for constitutively expressing fluorescent protein AmCyan (Figure D in S1 Text). AmCyanPH-1 conidia were produced in liquid mung bean broth (40 g mung beans per 1 L H_2_O) [39, 77] and resuspended in sterile water for inoculating maize plants within 2 h.

Maize (*Zea mays* ssp. *mays* L.) cultivar B73 plants [78], which are susceptible to *F*. *graminearum* causing *Gibberella* stalk rot [79], were cultivated in a phytotron at 22–26°C with 65% relative humidity and a 14 h photoperiod for 8 weeks until the tenth leaf appeared. Maize plants were inoculated by punching a hole in the stem at the second or third internode above the soil line using a sterile micropipet tip (10 mm hole depth), followed by injection of 20 μL macroconidia suspension at a concentration of 10^6^/mL. Mock-inoculated maize stalks treated with water served as the control. The plants were kept growing with the wounds being covered by sterile gauze to maintain moisture and avoid contamination from other organisms. At a given time point spanning 12 to 360 hai as indicated, the stalks of three plants were cut down, and the inoculated internodes were split longitudinally for symptom measurements. Each time point was repeated at least five times (independent experiments). The longitudinal length of brown infected areas was measured as the lesion size at the indicated time (illustrated in Figure D in S1 Text) using ImageJ software. The average distance from the wounding line to upside/downside front of the brown area was measured as half lesion size (illustrated in Fig 2A).

Half of the split internodes were further sliced longitudinally and/or transversely for microscopy (as illustrated in Figure B in S1 Text); the other half was saved for laser microdissection (see below). An Olympus BX51 microscope equipped with a green fluorescent protein filter set (450- to 480-nm excitation; 515-nm emission) and a differential interference contrast (DIC) module was used to take wide-field images, and the hyphal advance distances were measured based on images of longitudinal slices (as illustrated in Fig 2A). Confocal images were acquired on an Olympus Fv10i with two emission-collecting windows operating simultaneously. Excitation/emission wavelengths were 405 nm/460 to 500 nm for AmCyan and 405 nm/570 to 670 nm for plant cell wall autofluorescence.

For visualizing the plant secondary cell wall component lignin, longitudinal or transverse slices of mock-inoculated internodes were immersed in a phloroglucinol–HCl solution [80] for 5 min and pictured under a bright field. The intensity of phloroglucinol staining reflects the lignin content.

For visualization of host cell nuclei, longitudinal slices of the infected internode were stained with 4’,6-diamino-2-phenylindole (DAPI) solution (1 μg/mL with 0.2% [v/v] Tween 20) for 5 min before observation under a UV channel.

For testing host cell viability and/or plasma membrane intactness, cross sections of infected internodes were treated with 1 M NaCl for 10 min. Microscopic images of the same area were taken before and after treatment for plasmolysis assessment.

### Laser capture microdissection (LCM) and microarray hybridization

Pith tissues of the other half split internodes of mock-inoculated and *F*. *graminearum*-inoculated maize stalks at 12, 18, 36, 48, 72, 108, 132, and 144 hours after inoculation (hai) were fixed in acetone and embedded in paraffin as described [39] using a vacuum infiltration time of up to 30 min. Then, 12 μm-thick sections were obtained using a rotary microtome (Leica RM2235, Leica Biosystems Nussloch). A Veritas Microdissection Instrument (Acturus Bioscience) at the SIPPE core facility was used for LCM. The target fungal mycelia fluorescence-tagged with AmCyan were captured using the following settings: spot size 15–20 μm, power 95–100 mW, and pulse duration 3–4 ms. Targeted fungal hyphae were those invasively growing in pith tissues, not in rind or vascular bundle. In obtaining 72–144 hai samples, aerial hyphae in disintegrated tissues close to the wound site were also avoided. Approximately 500,000 μm^2^ of tissue was obtained for each sample. Two or three independent biological samples were obtained for each time point.

Approximately 10 ng total RNA in 11 μL volume was extracted from each laser captured sample using a Picopure RNA Isolation Kit (Arcturus Bioscience), and 1μL of the total RNA was used for quality assessment with an Agilent RNA 6000 Pico Kit and a Bioanalyzer 2100 instrument (Agilent Biotechnologies). Only those total RNAs with a 28S:18S ribosomal RNA peak height ratio >1 were kept for RNA amplification. Approximately 10 μg of biotinylated complementary RNA (cRNA) was generated from each total RNA sample using an Affymetrix Two-Cycle Target Labeling Assay Kit (Affymetrix) combined with a MEGAscript High Yield Transcription Kit (Ambion AM1333), as previously described [39]. Labeled cRNAs were hybridized to the Affymetrix *F*. *graminearum* genome GeneChip with ~14,000 *F*. *graminearum* genes presented [44]. Chip hybridization, washes, and chip reading were carried out with cRNA at ShanghaiBio Corporation according to the Affymetrix *Expression Analysis Technical Manual*. Three independent biological replicates were obtained for the time points 12, 36, 72 and 132 hai. Two independent biological replicates were obtained for mock inoculated sample and the time points 18, 48, 108 and 144 hai. Three biological replicates for spore suspension (0 hai) and three biological replicates for *in vitro* grown hyphae (72 h) were also obtained at the time of this work, as previously reported [39].

### Microarray data processing and analysis

Original CEL files were normalized by Robust Multichip Analysis (RMA) as described in [39]. Correlation coefficients between biological replicates (see Dataset A in S2 Text) for LCM-derived samples were approximately 0.95 (ranging from 0.93 to 0.99) based on RMA-normalized data. A presence/absence test was conducted with the detection quality P value < 0.004. A gene was considered to be present if the P value was < 0.004, or absent if the P value was > 0.065, in at least two biological replicates. To identify differentially expressed genes compared to *in vitro* grown hyphae, we used the web-enabled and cross-platform Significance Analysis of Microarrays (SAM) software package via Shiny [47] (http://statweb.stanford.edu/~tibs/SAM/). A false discovery rate of 0.05 was used as the cutoff value for statistical significance, and a two-fold change in expression was used as the cutoff for fold changes.

A total of 13,429 probe sets represent 13,429 unigenes from all 18,069 probe sets [39]. Eighty-three probe sets that were called present in mock-inoculated maize stalk samples were excluded to avoid cross-hybridization from maize stalk cell RNA in the laser-captured samples. Thus, 13,346 unigenes were chosen for subsequent enrichment and function analysis. Expression patterns in maize infection were identified based on the ratio of expression at eight time points during maize infection (12, 18, 36, 48, 72, 108, 132, and 144 hai) and in the spore (0 hai) versus *in vitro* grown hyphae (72 h). Results are shown in Dataset C in S2 Text.

To compare *in vitro* germination data with *in planta* expression data, CEL files of *F*. *graminearum* gene expression profiles obtained during conidia germination stages [81] were downloaded from Plexdb (http://www.plexdb.org/), and RMA normalization was performed on 45 combined microarray data sets at 13 time points (*in vitro* at 0, 2, 8, 24, and 72 h and in maize at 12, 18, 36, 48, 72, 108, 132, and 144 hai).

FunCat [48] annotations for *F*. *graminearum* genes were downloaded from FGDB ([49]). Out of 13,346 unigenes, 4965 had FunCat annotations. Using the χ^2^ test and P < 0.05 as the threshold for significance level, enriched FunCat families in maize stalk infection were delineated.

Genes involved in cell wall degradation (Dataset B in S2 Text) were manually annotated with reference to the Carbohydrate-Active Enzymes (CAZy; www.cazy.org) annotation for *F*. *graminearum* genes by Ma et al. (2010) [58]. SMB cluster genes (Dataset I in S2 Text) were obtained from [39, 55, 58].

Gene expression heatmap matrices were generated using the “heatmap” function of the amap package in the bioconductor software of R language software version 2.15.3 according to correlation coefficients calculated by a Pearson correlation algorithm. Principal component analysis was performed using library “scatterplot3d” in the “pca” package.

To compare transcription during maize stalk infection to wheat and barley head blight infection, gene lists from Supplemental Tables 1–5 in [72] were integrated with various gene lists generated in our work (see Datasets L, M, and G-J in S2 Text for results).

### Quantitative RT–PCR

The total RNA extracted from laser-captured samples was amplified to generate antisense RNAs using a TargetAmp two-round Aminoallyl-aRNA Amplification Kit (Epicentre Biotechnologies) and processed with an RNeasy MinElute Cleanup Kit (Qiagen). The cDNAs were synthesized using reverse transcriptase M-MLV (TaKaRa Biotechnology). Quantitative PCR experiments were conducted on a Bio-Rad MyiQ single-color realtime PCR detection system. The reaction mixture contained 5 μL of template (~20 ng), 10 μL of 2× SYBR green premix, 0.4 μL of forward primer (10 μM), 0.4 μL of reverse primer (10 μM), and 4.2 μL of nucleotide-free water. The reference gene was β-tubulin (FGSG_09530). Primer sequences are listed in Dataset O in S2 Text. Reaction efficiency was calculated from standard curves using a serial template dilution for each pair of primers. For specificity, only primers that generated a single peak in the melting curve were used (Figure Y in S1 Text). Each experiment included at least two biological replicates for each pair of primers and was repeated twice.

### BTA1-related *F*. *graminearum* strains construction

*BTA1* and other knockout mutants were generated using the split marker recombination method [82]. For complementation assays, a fragment containing the gene and promoter region was cloned into a vector containing a neomycin resistance cassette. The resulting constructs were transformed into protoplasts of the mutants. Mutants were verified as shown in Figure M in S1 Text.

For subcellular localization analysis, *BTA1* (FGSG_00742) cDNA was in-frame fused with mRFP in a hygromycin resistance containing vector, driven by the *F*. *graminearum* constitutive expression promoter *EF1-A* (*FGSG_08811*, translation elongation factor 1 alpha). An ER marker protein [65] fused with YFP, was inserted after the promoter *EF1-A* in a vector which confers neomycin resistance.

To generate *F*. *graminearum* strain constitutively expressing BTA1, *BTA1* cDNA was inserted into a hygromycin resistance containing vector under the control of the *EF1-A* promoter. Dataset O in S2 Text lists all the primer sequences.

### BTA1 heterologous expression in *E*. *coli*

For heterologous expression, *Fg-Bta1* cDNA harboring a C-terminal GST tag was cloned into vector pGEX-4T-3 (GE Healthcare) using a CloneExpress II One Step Cloning Kit (Vazyme). The resultant vector was transformed into *E*. *coli* BL21 cells; cells harboring plasmids with or without *BTA1* were grown in 2 mL overnight cultures, and then were inoculated into 200 mL of Luria-Bertani medium containing appropriate antibiotics. After growth to an optical density of about 0.6 at 600 nm, 0.2 mM isopropyl-D-thiogalactoside (IPTG) was added. Cultures were incubated at 18°C for 20 h and centrifuged to harvest for membrane lipid extraction.

### Membrane lipids extraction and DGTS detection

To determine the polar lipid composition, *F*. *graminearum* hyphae grown on phosphate limited media for 14 days and *E*. *coli* harboring the BTA1 expression vector grown after IPTG induction for 20 h, respectively, were harvested (~40 mg). Lipids were extracted according to [83], and then spotted on thin layer chromatography plates (TLC Silica gel 60 F254; Merck), resolved with chloroform-acetone-methanol-acetic acid-water in the ratio 10/4/2/2/1, and visualized with iodine vapor.

The spots corresponding to a DGTS standard sample were scraped from the TLC plates, eluted with chloroform, and then concentrated under a stream of nitrogen. The lipid extracts were dissolved in CHCl_3_/methanol/50 mM sodium acetate in water in the ratio 300/665/35. 1,2-dipalmitoyl-sn-glycero-3-O-4'-(N,N,N-trimethyl)-homoserine (Avanti 857464) was used as external standard DGTS.

Samples were further analyzed using the Agilent G6520A accurate-mass Q-TOF LC-MS system with an Eclipse Plus C18 column (4.6 × 250 mm, 5 μm). The injected volume was 10 μL with a flow rate of 0.3 mL/min. A was water + 20 mM NH_4_OAc. B was methanol, and the gradient profile was from 0 min 65% to 15 min 85%, 25 min 100%, 28 min 100%, 40 min 65%. The mass range was 400 to 1500; nebulizer pressure 40 psig; drying gas N_2_ 350°C, 9 L/min; ESI Vcap 3500 Vl fragmentor 214 V; skimmer 65 V; and Oct RF Vpp 750 V. DGTS was analyzed as [M+CH3COO]^−^ ions, had a UV peak at ~25 min retention time, and its corresponding MS peak appeared in the extracted ion chromatograph with a mass-to-charge ratio 770.610, (DGTS formula: C_42_H_81_NO_7_, calculated accurate-mass for M+CH3COO^−^: 770.6152; measured mass: 770.610; error: 6.75 ppm). DGTS was further confirmed by matching measured versus predicted isotopic distributions of masses 770.61, 771.61 and 772.61 (Fig 8).

### Virulence assays of maize

For virulence assays in maize stalks, fully grown maize plants were inoculated as described above in the session of maize stalk infection and microscopic observation, with indicated strains. At 7 dpi, the stalks were split longitudinally and the symptoms were documented by photography. The extent of the lesion area was quantified using ImageJ software version 1.46 (http://rsbweb.nih.gov/ij/index.html). Statistical significance was determined by a Student’s *t*-test (P < 0.05) as implemented in GraphPad Prism 5 software. At least five internodes were inoculated at a time; inoculations were repeated five times for each fungal strain.

KH_2_PO_4_ was added to distilled water to final concentrations of 0.1 M, 0.01 M and 0.001 M, respectively, at pH 6.8. At 8 h after inoculation by *bta1* mutant M1, 10 μL KH_2_PO_4_ solution were added into the wound site of maize stalk using a sterile micropipet tip.

### Determination of phosphorus content in maize stalk fractions

Stalk internodes of 8-week-old maize B73 plants were fractioned into xylem sap, apoplastic fluid and stalk tissues (Figure K in S1 Text). The xylem sap collection method was modified from previous reports [84, 85]. After the root system was removed, stem internode sections about 6 cm in length (roughly 6 g) were used. A 5-mL syringe filled with sterile distilled water was jointed to the lower end of the stem and the juncture sealed tightly with parafilm. Hydraulic pressure was applied slowly using the water-filled syringe and sap was collected in a tube.

After removing the xylem sap, the 6 cm-long internodes were cut equally into two parts. One-half was used for apoplastic fluid collection using the method described in [86]. The half maize stalk was cut into flakes and put in a beaker with 100 mL water. The beaker was placed in a desiccator jar and vacuumed for about 30 min. After removing excess water, the flakes were put into a filtering centrifuge tube and centrifuge at 3000 × g for 10 min at 4°C. After centrifugation, about 1 mL of apoplastic fluid was extruded from the stalk. The other half internode was ground into powder in liquid nitrogen to obtain the total fluid of maize stalk as a control. The same vacuum-centrifugation method as above was used to collect apoplastic fluid from wheat coleoptiles.

Phosphorus content was analyzed by inductively coupled plasma mass spectrometry using a PerkinElmer ELAN DRC-e ICP-MS at the Shanghai Institute of Plant Physiology and Ecology Core Facility for Mass Spectrometry, according to [87] and [88]. Tissue extracts were digested overnight in concentrated nitric acid at room temperature and diluted with deionized water to a final volume of 7 mL. Aqueous standards at 125, 250, 500, and 1000 ppb phosphorus, purchased from Shanghai Institute of Measurement and Testing Technology (Shanghai, China) were used to generate a calibration curve. Phosphorus content was determined as a mono-isotopic element at mass 30.9938 atomic mass unit.

### DON detection

DON concentration was measured by ELISA using the RIDASCREEN FAST DON Kit (R-Biopharm AG, Germany) [89]; its determination limit is 0.2 ppm. A calibration curve was established using 0, 0.222, 0.666, 2 and 6 ppm DON. For sample preparation, 1 g of maize tissue was ground and extracted in 20 mL of distilled water. After shaking vigorously for 3 min, the solution was filtered through a Whatman No. 1 filter and the filtrate was used for further analysis following the kit manufacturer’s protocol. Finally, after addition of stop solution, the absorbance at 450 nm was measured on an Eon Microplate Spectrophotometers (BioTek, USA). Experiments were repeated twice.

### Accession numbers

XM_380918.1 for FGSG_00742 (BTA1), XM_382253.1 for FGSG_02077 (CFEM1), XM_011327926.1 for FGSG_06610 (PhoD), XM_011324063.1 for FGSG_03366 (PHO12), XM_011329171.1 for FGSG_07678 (PHO 610), XM_011324021.1 for FGSG_03402 (PHO11), XM_391412.1 for FGSG_11236 (PLC), XM_011323534.1 for FGSG_03846 (TAGL), XM_011324206.1 for FGSG_03243 (TAGL), XM_011329299.1 for FGSG_07783, and XP_389706.1 for FGSG_09530 (tubulin).

The microarray data have been deposited in NCBI's Gene Expression Omnibus [90] and are accessible through GEO Series accession number GSE53854.

## Supporting Information

S1 Text Supplemental figures. (PDF)

S2 Text Supplemental data. (XLSX)
