# Supplementary material for: Cellular Tracking and Gene Profiling of Fusarium graminearum during Maize Stalk Rot Disease Development Elucidates Its Strategies in Confronting Phosphorus Limitation in the Host Apoplast
Source: PLoS Pathog. 2016 Mar 14;12(3):e1005485. doi: 10.1371/journal.ppat.1005485 (PMC4790934; doi:10.1371/journal.ppat.1005485)
Supplement: S1 Text — (PDF) [file ppat.1005485.s001.pdf]

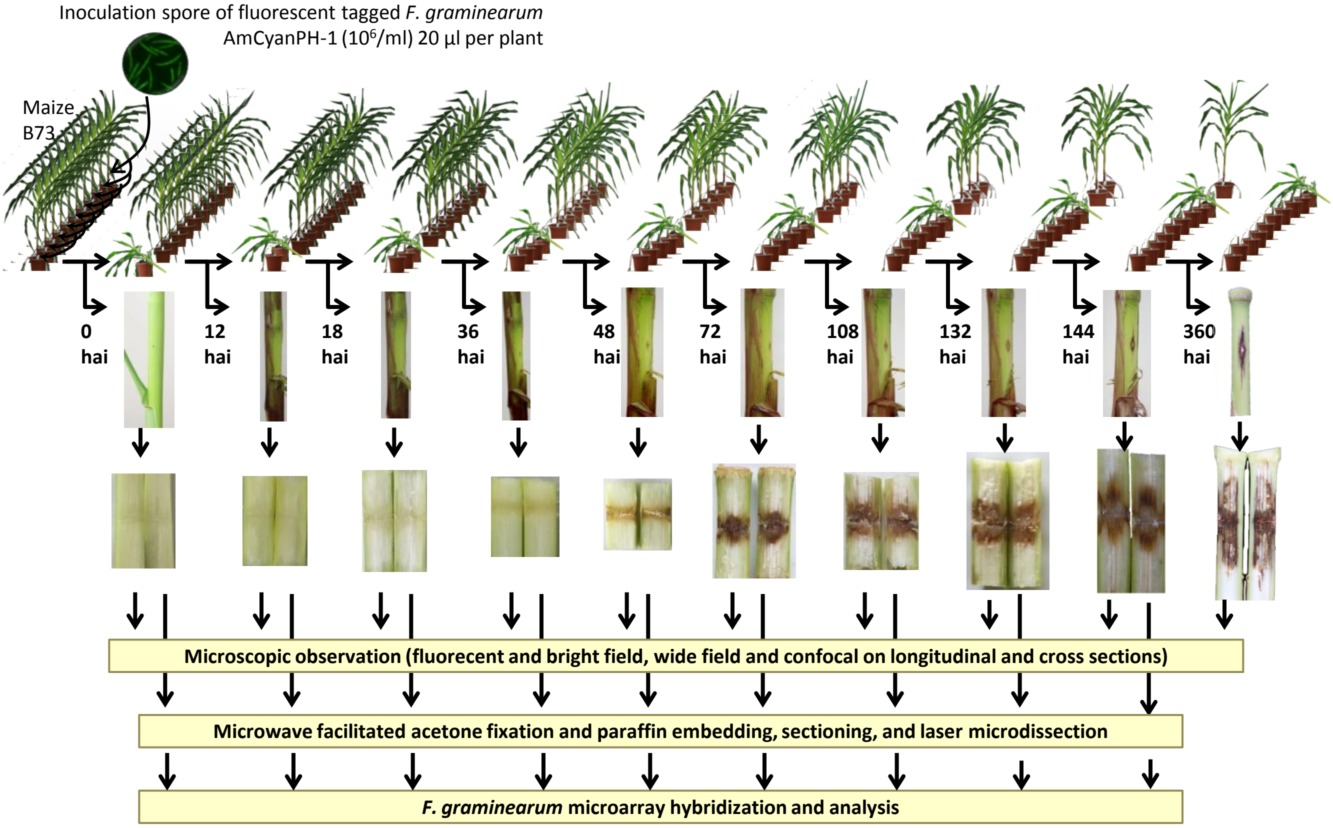

Figure A. Experimental design of this work.

hai: hours after inoculation. For simplicity, one plant per time point is shown, while in actual experiments 3 plants per time point were used for each experiment, and the experiments were repeated five times. To show the surface lesion development in a continuous way, the pictures of intact stalk internodes from 12 to 360 hai were taken from one plant, while the split internode pictures were taken from different plants.

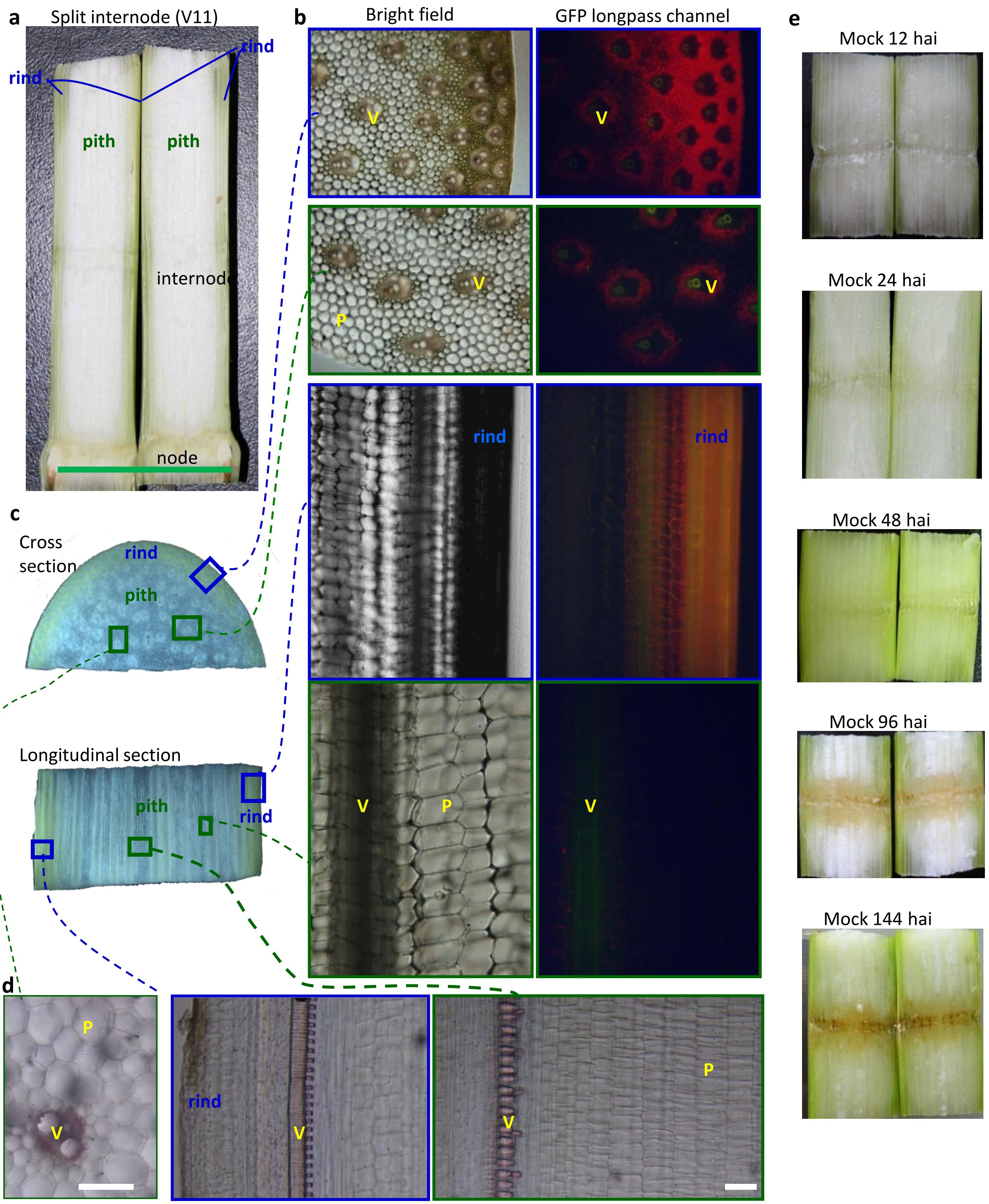

Figure B. Anatomy of maize stalk.
Uninfected internodes of maize stalk at V11 stage are shown as reference for understanding *F. graminearum* progression. V: vascular bundle. P: parenchyma cells. The bottom panel pictures (d) were stained by phloroglucinol in which lignin exhibiting pink. (e) mock inoculated internodes splitted at indicated time after inoculation. Hai: hours after inoculation. Green bar=5 cm, White scale bar =100 µm.

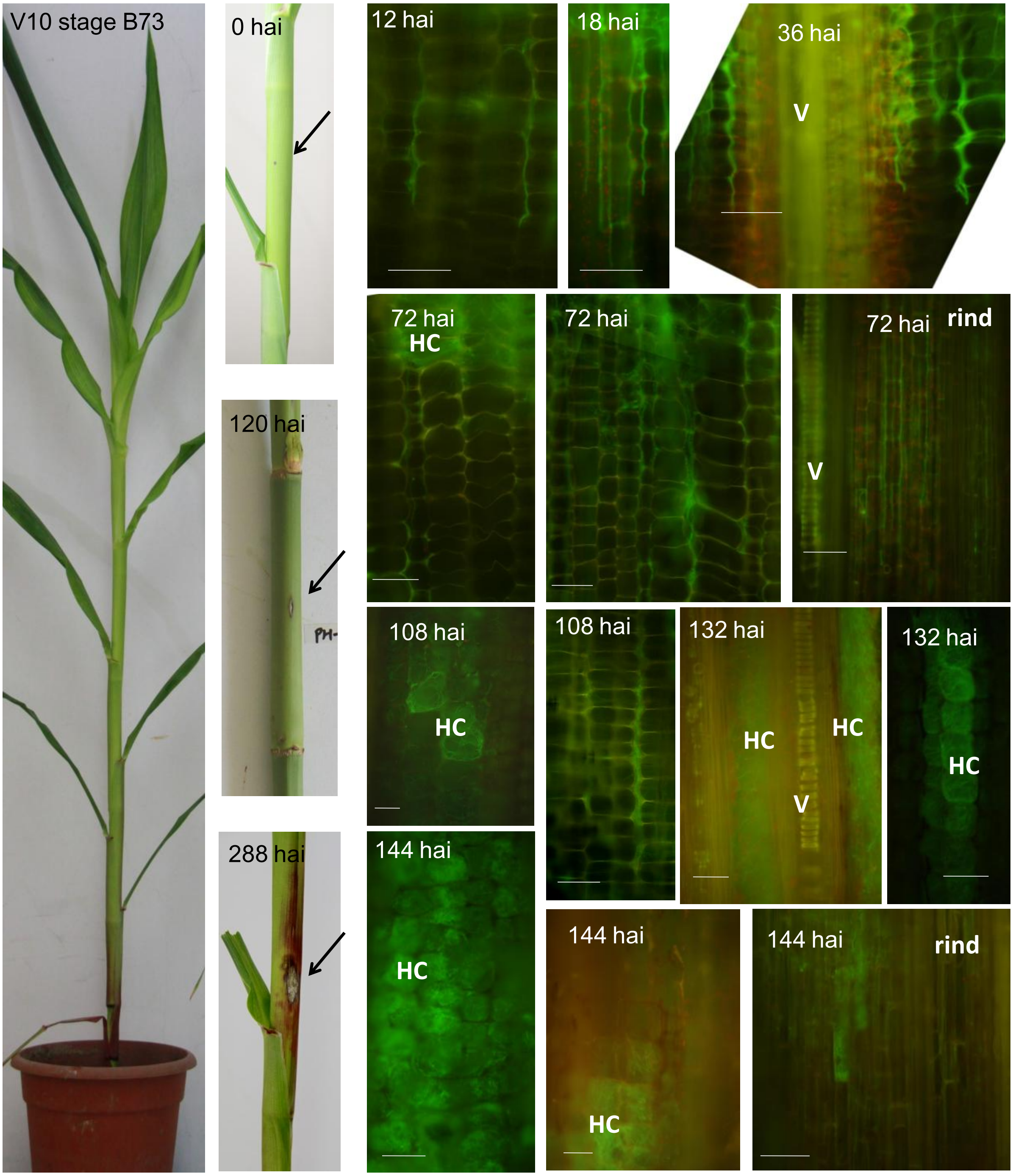

Figure C. Additional pictures of maize stalk infection.
Maize stalks inoculated by AmCyan expressing *F. graminearum*. The pictures show pith tissues except for those labeled as rind. V: vascular bundle. HC: host cells fully occupied by fungal hyphae. Scale bar = 100 μm. Note that 36 hai image clearly shows that multiple hyphae longitudinally growing along parenchyma cells besides a vascular bundle without entering it, unlike those wilt fungus such as *Fusarium oxysporum*, suggesting *F. graminearum* not prefer to take route of vascular bundle. Also note that in the left 132 hai image, many fungi have occupied the space beside a vascular bundle and beside rind fiber cells, but have not colonized the rind fiber cells and vascular bundle cells. The three 144 hai images were from different areas of a maize stalk at the same time, the three images of 72 hai were from different areas of a maize stalk at the same time, both showing a slower progression in fiber cell areas than in parenchyma cell areas.

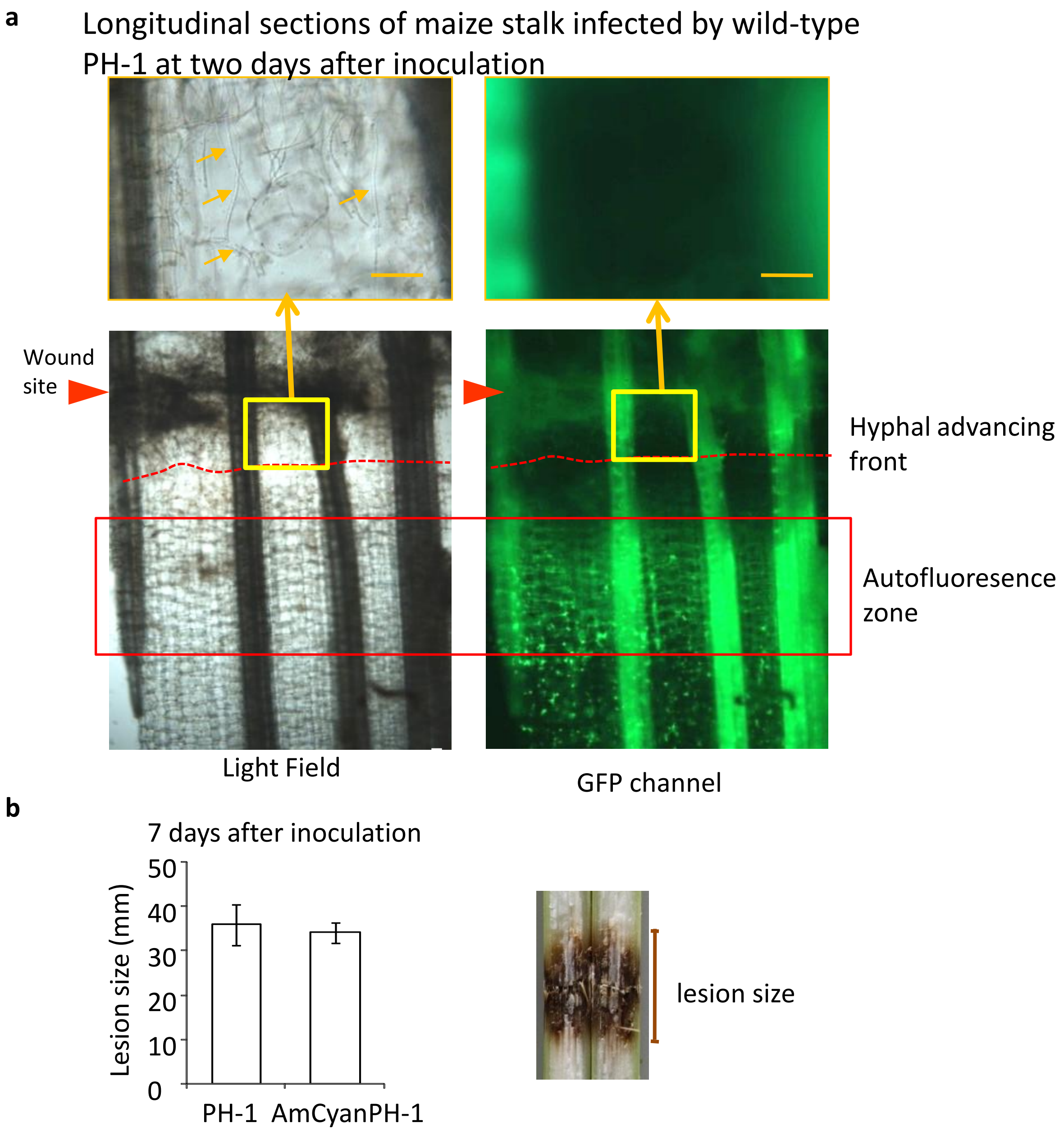

Figure D. *F. graminearum* AmCyanPH-1 strains behave similarly to wild type.
(a) Autofluorescence zone of maize stalk cells ahead of fungal hyphal arrival, inside the stalk infected by *F. graminearum* PH-1. Arrows point to fungal hyphae. Comparing to Fig S2B right, note the strong autofluorescence was an effect of *F. graminearum*. Scale bar = 100 µm.
(b) Lesion lsize measured at 7 days after inoculation.

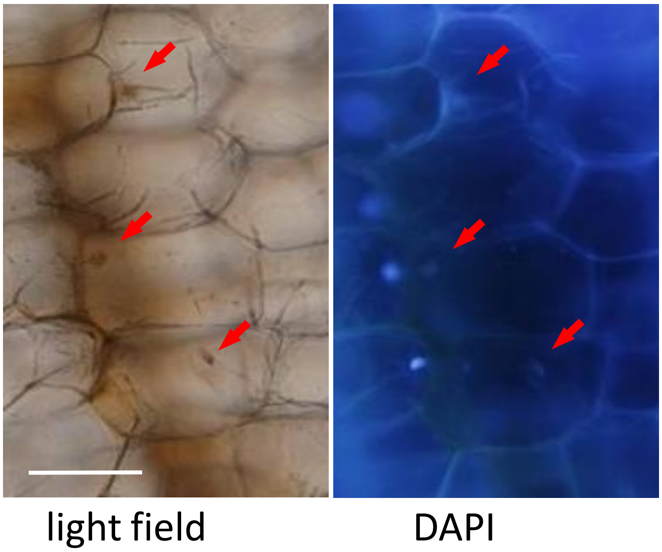

Figure E. DAPI-staining of parenchyma cells.
The pictures were taken at 120 hai of the maize stalk infected by *F. graminearum*. Arrows point to debris-like structures that can be weakly stained by DAPI. Scale bar = 100 µm.

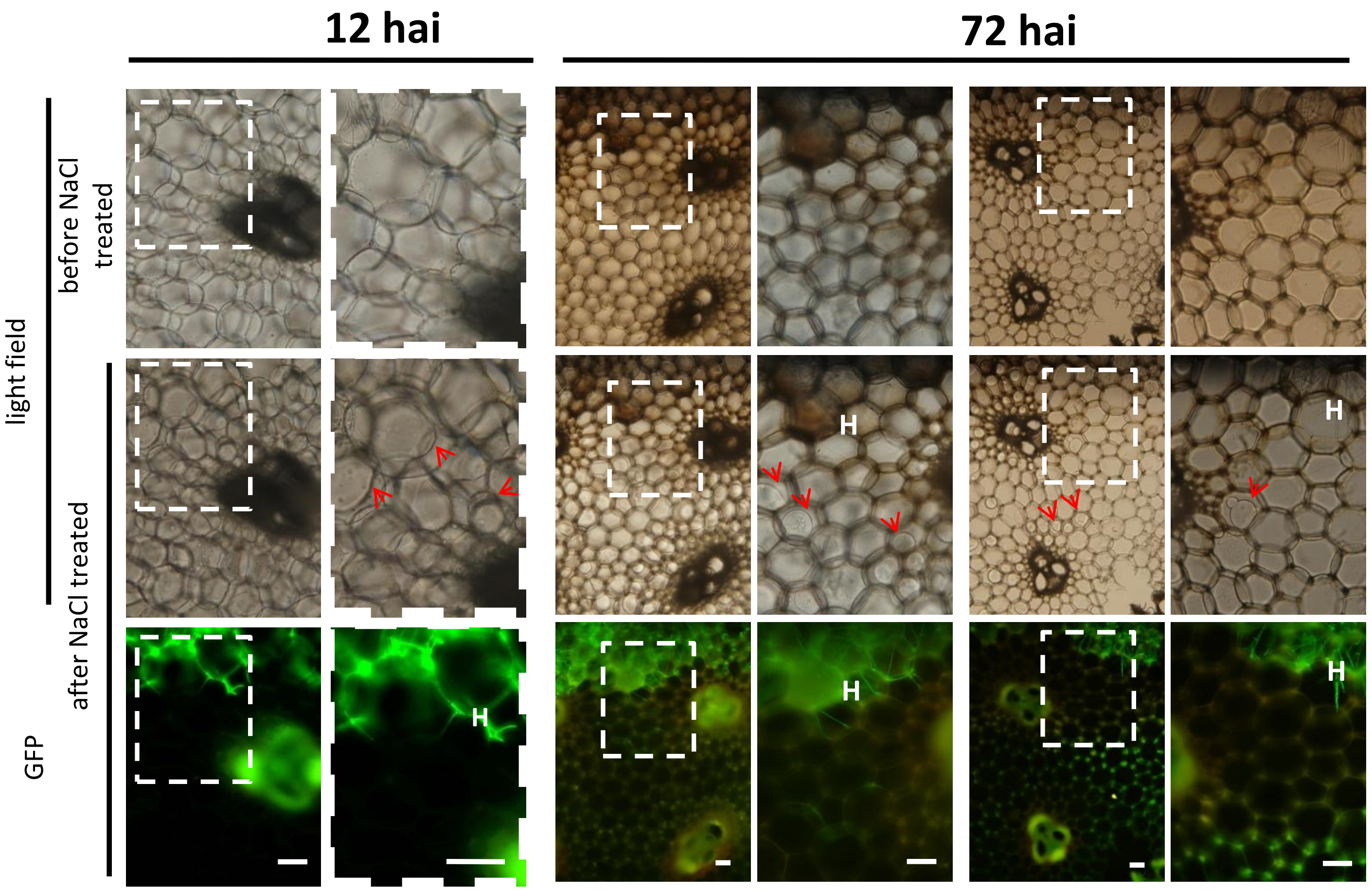

Figure F. Additional images of plasmolysis assays. Maize parenchyma cells before and after 1 M NaCl treatment for 10 minutes are shown. *F. graminearum* AmCyanPH-1 hyphae are visible as green lines under GFP channel. Red arrows point to maize cell membranes away from cell walls. H: hyphae. White scale bar = 100 µm. Images in this Fig were taken from cross sections. hai: hours after inoculation. Note that at 72 hai, the cell undergoing plasmolysis is away from the hyphal reached cell.

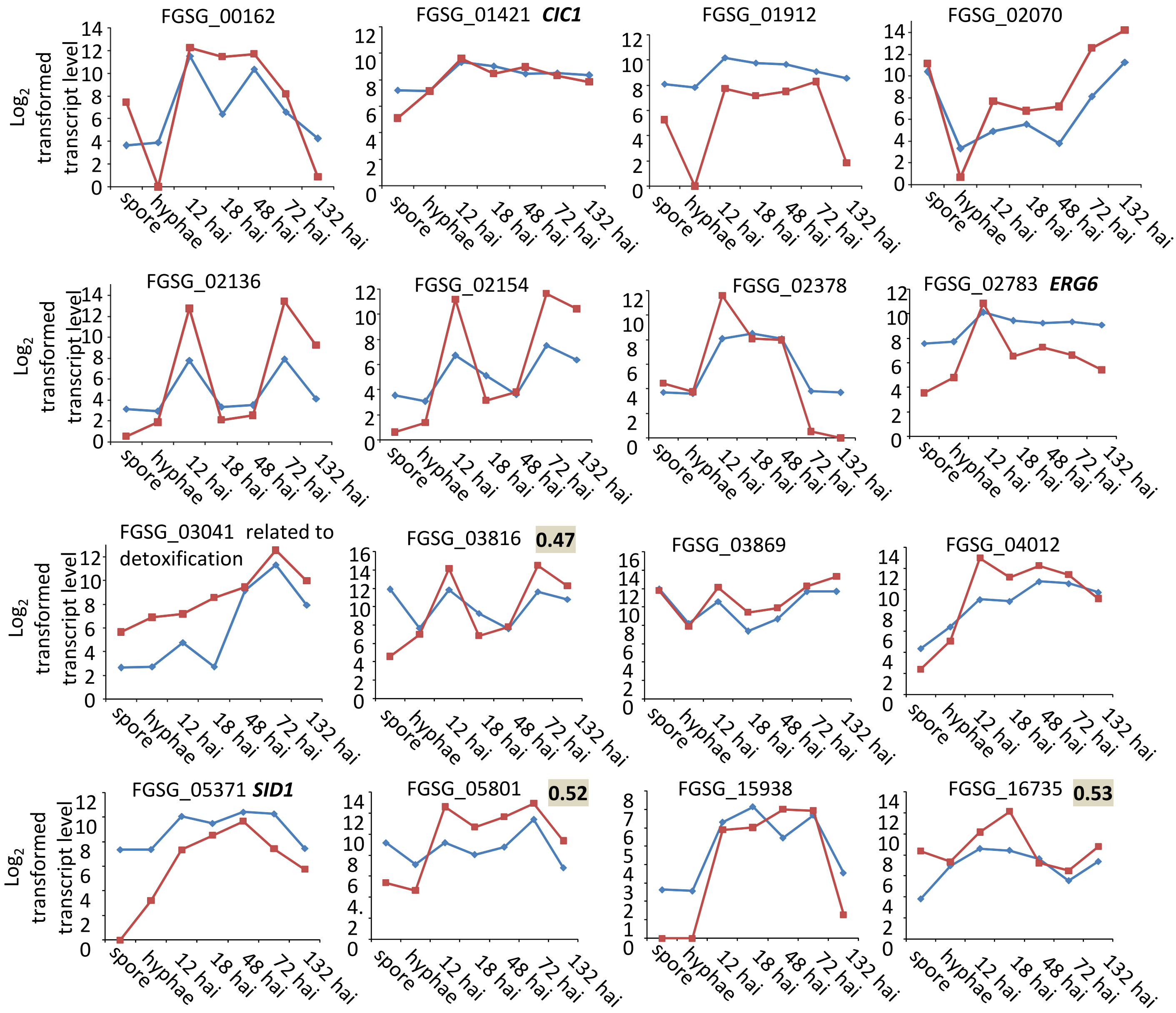

Figure G. Quantitative RT-PCR analysis 16 *F. graminearum* genes.

Gene expression at 7 time points were examined by quantitative PCR after reverse transcription. Red lines showed the qRT-PCR results, and the blue lines showed data from microarray hybridization. hai (hours after inoculation for maize stalk). The values of quantitative RT-PCR was scaled according to microarray data of the reference gene. Among the 16 genes, expression of 13 genes were highly consistent between microarray data and qRT-PCR (r>0.8), 3 were not consistent (r<0.75).

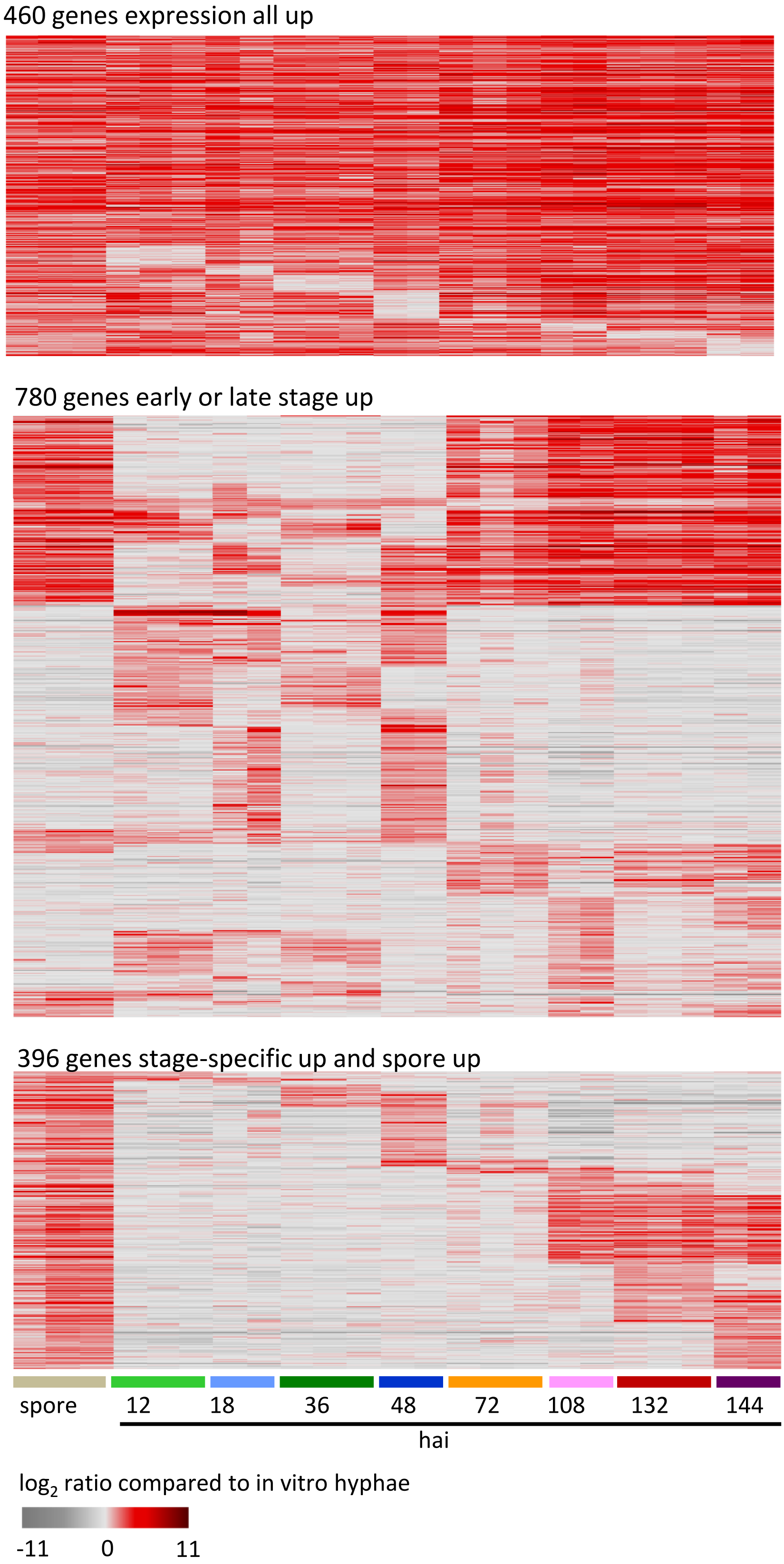

Figure H. Additional heat maps of genes that were significantly up-regulated in maize stalk infection compared to *in vitro* hyphae.

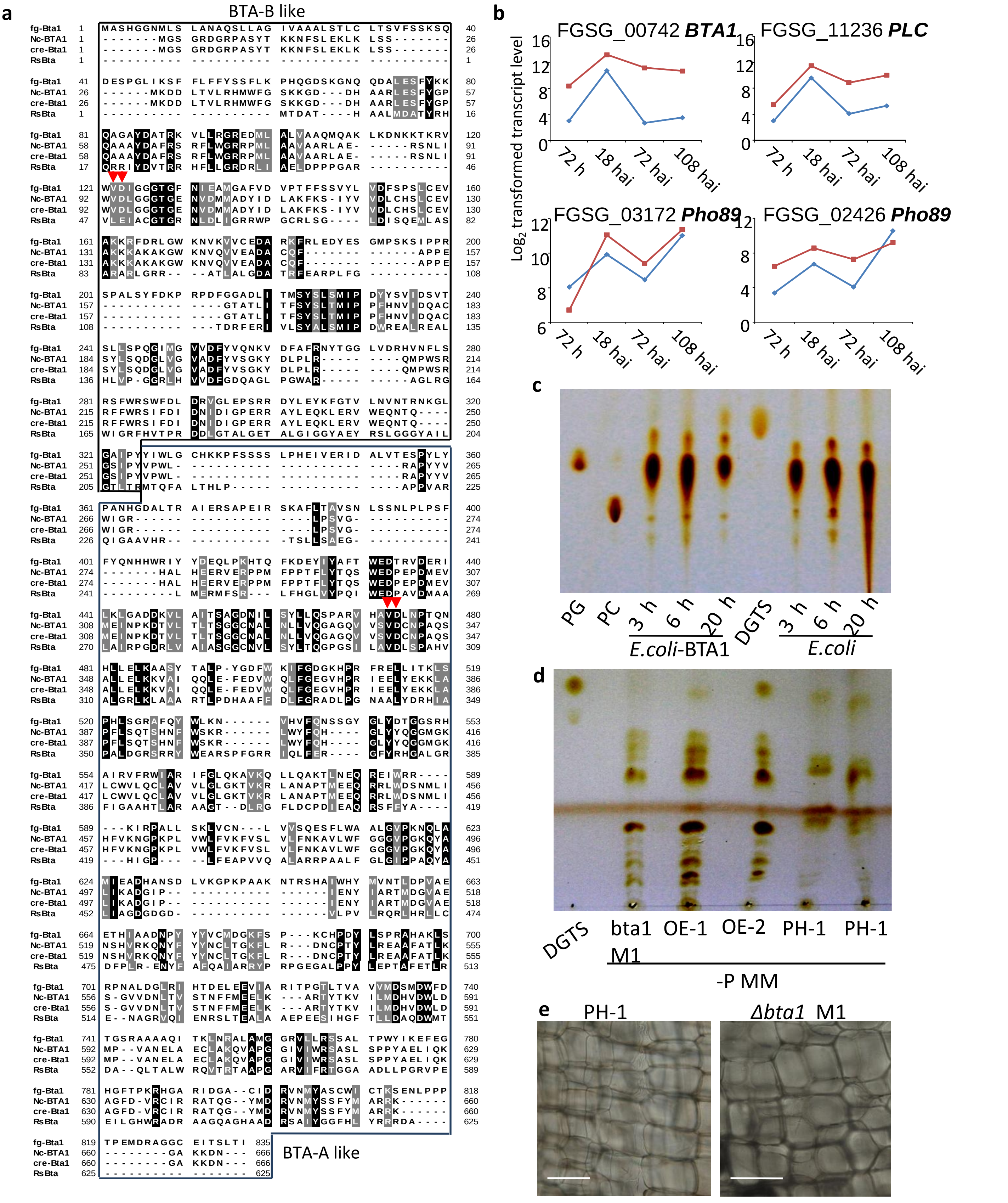

Figure I. Sequence and function analysis of *BTA1*.
(a) Amino acid sequence alignment. Fg: *F.* graminearum; Nc: *Neurospora crassa:* Cre: *Chlamydomonas reinhardtiii* ; Rs: *Rhodobacter sphaeroides.* Red arrow heads point to the residues critical for AdoMet binding. BTA-B like and BTA-A like domains are boxed. **(b)** qRT-PCR. Red lines show the qRT-PCR results, and the blue lines show data from microarray. **(c) (d)** TLC analysis of lipids extracted from *E. coli* or from *F. graminearum* grown on media without phosphate (-P) (-P) . OE-1, OE-2: BTA1 overexpressed strains. MM : minimal medium. **(e)** The original images of Fig 7C (6 days).

a >FGSG_03846

MAIHRVLALLSLWLGLVAAIPSSHIYKRSESSQPLPPSEDPWYSAPDGYEDEEPGTVLRLRPAPGNLTSIVGNCSAI
YNILYRTTDSQYKASWAVTTLYVPKLGSNSSAARVFNQSAVISYQAPYDSADVDASPSYTAYSSGGTDLFNLALGL
GVFLNVPDYEGPLASFTAGVISGHATLDSIRAVLSLGLGLNTESPRVALWGYSGGALASEWASELAVQYAPDLQE
SVVGAAIGGITPNITAVVESISGKDAAGLGPSGIIGITSQYPEVQKYVISKLKTEGPQNKTGFLAVKGFTVQEAGAA
YAGVNIFDFFKDGMNLFRDPKILKVINRDGIMGYHGVPQWPIFAYQAVHDEISPIANTDKLIERYCAVGANILYQ
RNSVGSHSEEFYLSAAPAIQWLAAVLTGQYASVYKTEGCTIQNVTRNSTAIPLKKRNAPNGVFNLW

>FGSG_03243

MMPSLLSLVTAAALLVEGVVSQQTPTVKVKNGTLEGRYLPGYNQDLFLGIPFAQPPVGQLRFQNPQSLNETFDT
LKVKKYGDSCVGYGNSADQGPATFSENCLTLNVVRPAGTSKNAKLPVGLFIHGGGWTMDFAANGAYNMTFM
VEEAAKAGKPFVAVSIAYRLAFWGFMASKDIMDAGVANLGLKDQRLAMHWVKENIGAFGGDASKVTIFGESA
GGGNVGYHATAYGGRDDKLFRGIIAESGADGTDMKNLTEPDRRYNIIAEAAGCGDSSDKLACLRKVPFKKLNAT
STKVPGNFYPVVDYDFIPDYPSILLENGKFVKVPLMAGTNADEGSFFALPGVDTDEQVAAAIMAGGVDANTTE
TLMALYPNIDALGIPSGYRRKAGDPVKAQFKRMAAFQGDNLFVSWRRRRSDAWSKYGVPVYTYLFESPNKNM
PAYIGTPHFVEVAYVFFNKFGMGYGKNQGPLYNASKEVLDLAQLVSRMWISFITEGNPNEHGLPGWPKWPVN
KNGGGYGENFYFNPNGSSVQPDTFRLAGTAYMNSLASEQFGR

b PhoD

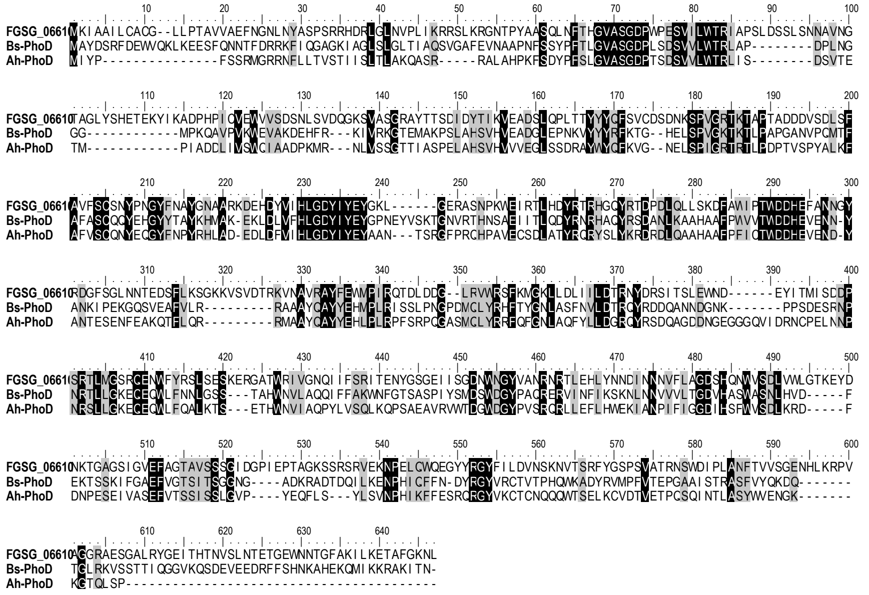

c

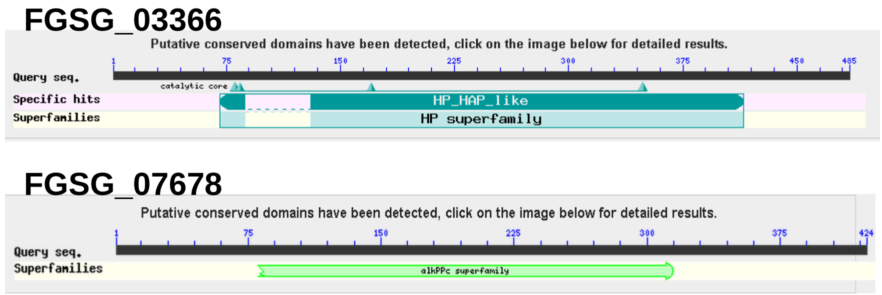

Figure J. Sequence analysis of putative lipase and phosphatase genes.
(a) Sequence analysis of two triacylglycerol lipase. Signal peptides are underlined and esterase lipase conserved domains are highlighted in purple based on conserved domains searched in NCBI. **(b)** Amino acid sequence alignment shows that *F. graminearum* FGSG_06610 is similar to *Bacillus subtilis (Bs)* PhoD and *Aphanothece halophytica (Ah) PhoD.* **(C)** Conserved domains in FGSG_03366 and FGSG_07678 using sequences of amino acid to blast in NCBI. HP: histidine phosphatase, alkPPc: alkaline phosphatase.

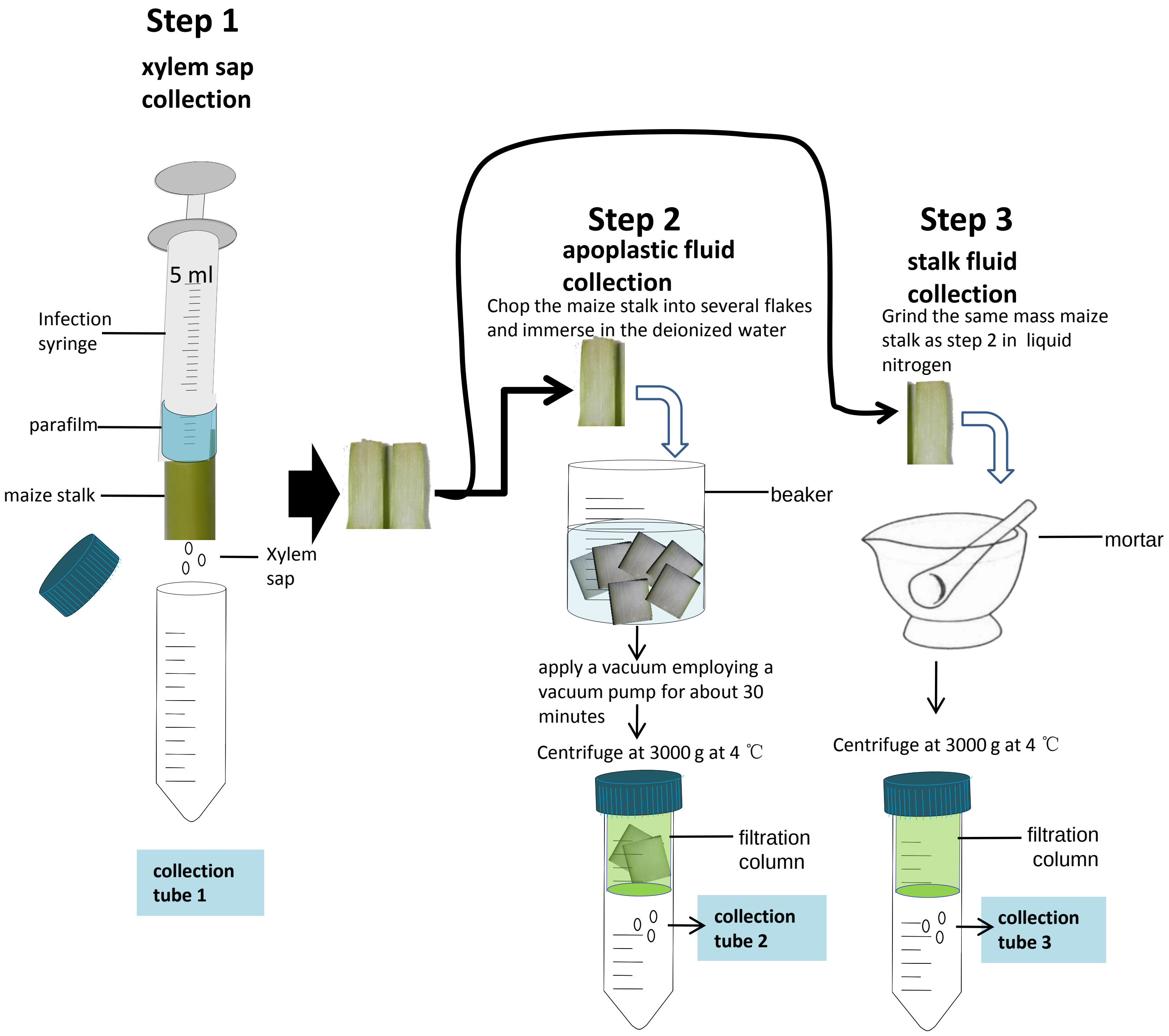

Figure K. Schematic diagram of maize stalk apoplastic fluids collection.
The method follows description in [88, 89] with slight modification adapt to maize stalk sample. The sample prepared for phosphorus contents determination by ICP MS.

88. Alexou M, Peuke AD (2013) Methods for xylem sap collection. Methods in molecular biology 953: 195-207.
89. Joosten MH (2012) Isolation of apoplastic fluid from leaf tissue by the vacuum infiltration-centrifugation technique. Methods in molecular biology 835: 603-610.

a

Phosphorus content in fractions of maize stalks at V10 stage. (corresponding to Fig 6D)

	**Maize stalk**	phosphorus (µg/g fresh weight)		
		apoplastic fluid	xylem sap	stalk
**Exp 1**	biological rep 1	2.27	4.27	92.17
	biological rep 2	4.62	8.35	123.67
	biological rep 3	3.22	6.81	97.18
**Exp 2**	biological rep 1	2.64	6.29	147.93
	biological rep 2	4.46	3.93	162.17
	biological rep 3	1.98	5.52	130.67
**Exp 3**	biological rep 1	2.80	4.33	110.13
	biological rep 2	1.87	3.84	144.67
	biological rep 3	1.13	–	145.83
	Mean ± SE	2.8±0.5	5.3±0.8	132±17
	estimated concentration (mmol/L)	0.34		3.19

b

Phosphorus content in maize stalks inoculated by water (mock) or *F. graminearum*

**maize stalk**		Phosphorus (µg/g fresh weight)	
		xylem sap	apoplastic fluid
**mock**	Exp 1- Ave	4.95	1.24
	Exp 2- Ave	4.52	2.78
	Exp 3- Ave	6.47	4.16
	Mean ± SE	5.31 ±1.03	2.72 ±1.46
	Estimated concentration (mmol/L)		0.33
**18 hai**	Exp 1- Ave	3.81	1.13
	Exp 2- Ave	3.09	1.55
	Exp 3- Ave	5.20	1.86
	Mean ± SE	4.03 ±1.07	1.51 ±0.37
	Estimated concentration (mmol/L)		0.18
**144 hai**	Exp 1- Ave	5.46	1.95
	Exp 2- Ave	7.01	1.96
	Exp 3- Ave	6.92	2.15
	Mean ± SE	6.46 ±0.87	2.02 ±0.11
	Estimated concentration (mmol/L)		0.24

c

Phosphorus contents in coleoptiles of 3 days-old wheat seedlings

**Wheat coleoptile**		phosphorus (µg/g fresh weight)	
		apoplastic fluid	whole coleoptile
**Exp 1**	biological rep 1	8.58	193.55
	biological rep 2	9.66	176.05
	biological rep 3	5.53	215.60
**Exp 2**	biological rep 1	5.85	220.85
	biological rep 2	5.88	246.75
	biological rep 3	5.29	162.40
**Exp 3**	biological rep 1	10.89	203.35
	biological rep 2	8.82	254.10
	biological rep 3	11.59	240.10
	Mean ± SE	8.0 ± 2.4	212.5 ± 18.8

Figure L. Phosphorus measurements in maize stalks and wheat coleoptiles. ICP-MS measurements of phosphorus contents in various fractions of maize stalks(a, b) and wheat coleoptile (c). hai: hour after inoculation by *F. graminearum*.

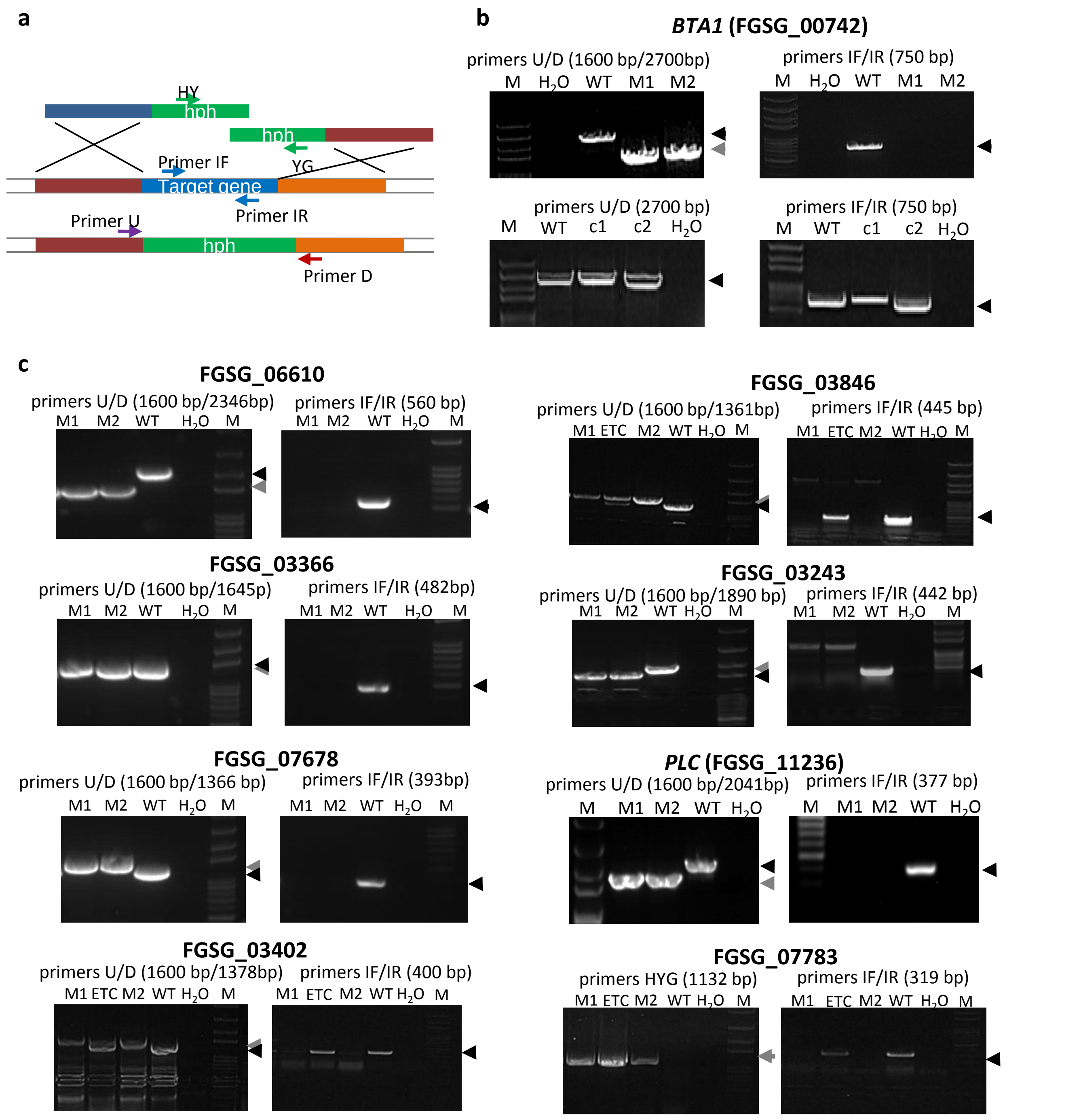

Figure M. Verification of knockout mutants and complemented strains.
(a) Construction of knockout stains by split marker recombination. **(b-c)** Verification of gene deletion mutants by genomic DNA PCR. Expected sizes of PCR products using the denoted primers are provided in parentheses. For those using primers U and D, sizes of hph inserted templates are provided before the size of wild-type templates. Black arrowheads indicate PCR products of wild-type gene, grey arrowheads indicate PCR products of the null mutants. The primer location on the target fragment are shown in **a**. Hph, hygromycin phosphotranferase gene cassette that confers hygromycin resistance; M, marker; WT, wild-type PH-1; M1-M2, mutants; C1, C2 represent independent complemented lines of M1. ETC: ectopic inserted strain.

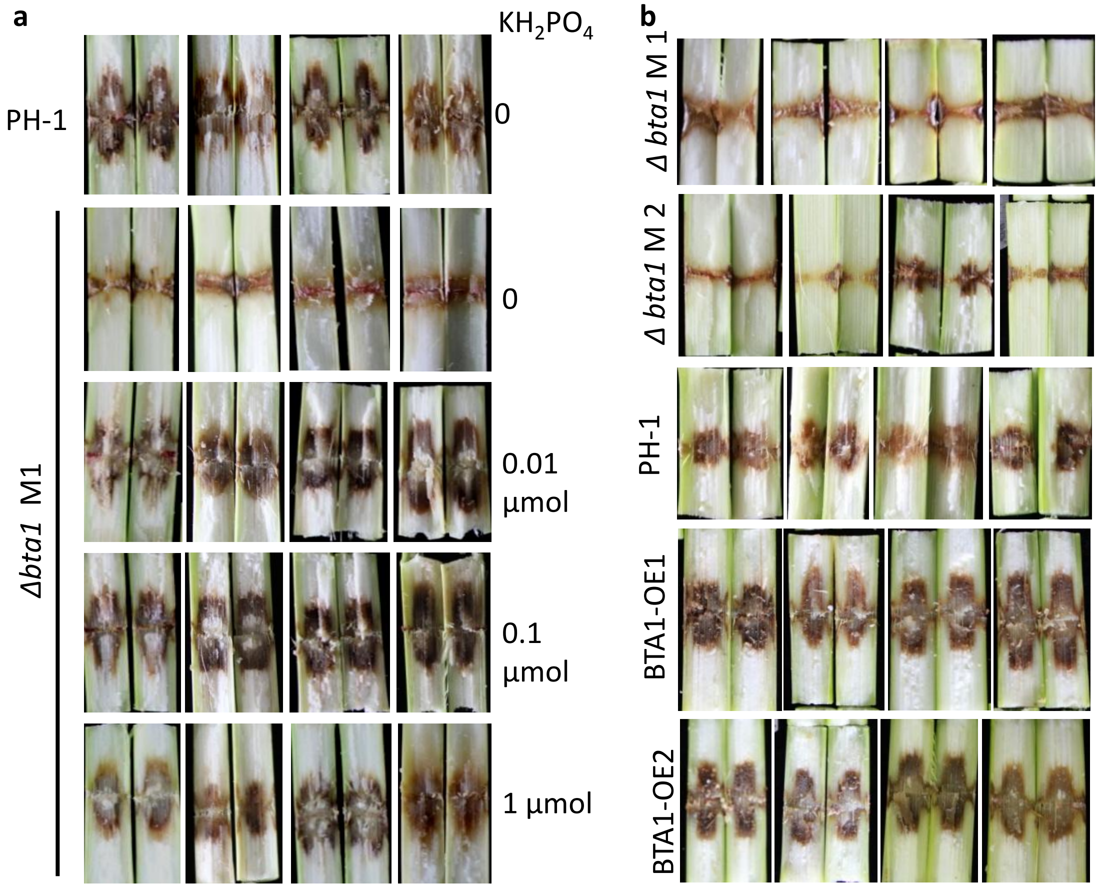

Figure N. Virulence assays of Δ*bta1* and BTA1 constitutive expressing strains. **(a)**Virulence assays on maize stalk supplement with KH_2PO_4 , KH_2PO_4 were added 8 hours after spore inoculation. **(b)** Virulence assays of Δ*bta1* and BTA1 constitutively expressing strains on maize stalks. Lesions were measured at seven days after inoculation.

>gi|758213871:2433009-2434008 *Fusarium graminearum* PH-1 chromosome 1, whole genome shotgun sequence FGSG_00742 promoter 1kb

GTAGCTGTGCATTTAGCATCACCAATTGACCA CAAGTG AAGATACCCCAATAGAAACAACAAAACACCATCA
CAACCGATAAATCCCGTGCGACTAAATTGGTCTTTTCTTTGCCGCCACAAAGGACCTCTAATATCTATACTCAC
TGCAATCTTGGTGGCTGTTGTAGGTGCCCTTACACTGGAGTTGATTGCTG CATGTGGTGGGCTGCTCTACTCT
GATTGGTGCTTGAACTTCTCATCTGGGACAACCGACTAACACGAAAATTCCAACCATGCTCAACCCAAACAA
CATCACAGCATGTTACTCTATTCCATGAGTATATTTGGAGTCATTTGGCTTCTCTTCTCTCT CATGTGAAGAGCC
CCTCAATCCGACAGAGTACTGTCATAATTGAGGGTTCAGCTTCTCTCAACACCAAACCGTGCCCAAAGACCG
TCAACTCCTTCCACTGTACAAGAACAAGATCGTCGTCTGTATTGAGTTGAGACCAGTACTAATCTATACCCCAC
TCACACGCTGTATAGAACGTCAACTCTTCTTAGCGAAC GAAGGATGGCACATGTCTGCCGCCGCCGTAGCCT
TGCAAGACCCCTAGAACTATACCTACCATGAA CATGTGCTTCACTTATTGGCTACCATCCCCATTACCCAACTC
CCGG CTCGTG CCTAGAATGAACACTGATGGCGGGGTTATCATGACTATCTGGTGGAGTCAAGAAGATCAACA
GGGCCTTGCAGACAGACG CAAGTG ATTTCGCGTCAAGTATTTCATGTTTGAGGCGTTATGGCCGCGCCGCC
GAGAT CATTGCTACCGAATTGAGTCCCAAATTAACTGGTGTAGAATTGAATTCTCCTGCATATATATCCCTAGC
CTGTCCTGTTTTCGTTCCCTGAATTCTTCTCTATCTCAAACCTGATCGTGTGAACTGCTTTCAACACAGCTTAC
AAGCCTCCTCCTTTCAACACTTATTCTCTCAGCATACAATGAGAGAGTACC

Sequences similar to “CACGTG” phosphate starvation response element are highlighted.

Figure O. Phosphate starvation response elements in the *BTA1* promoter.
Element sequence is based on Peleg et al., 1994.
Peleg Y, Metzenberg RL (1994) Analysis of the DNA-binding and dimerization activities of *Neurospora crassa* transcription factor NUC-1. Molecular and cellular biology 14: 7816-7826.

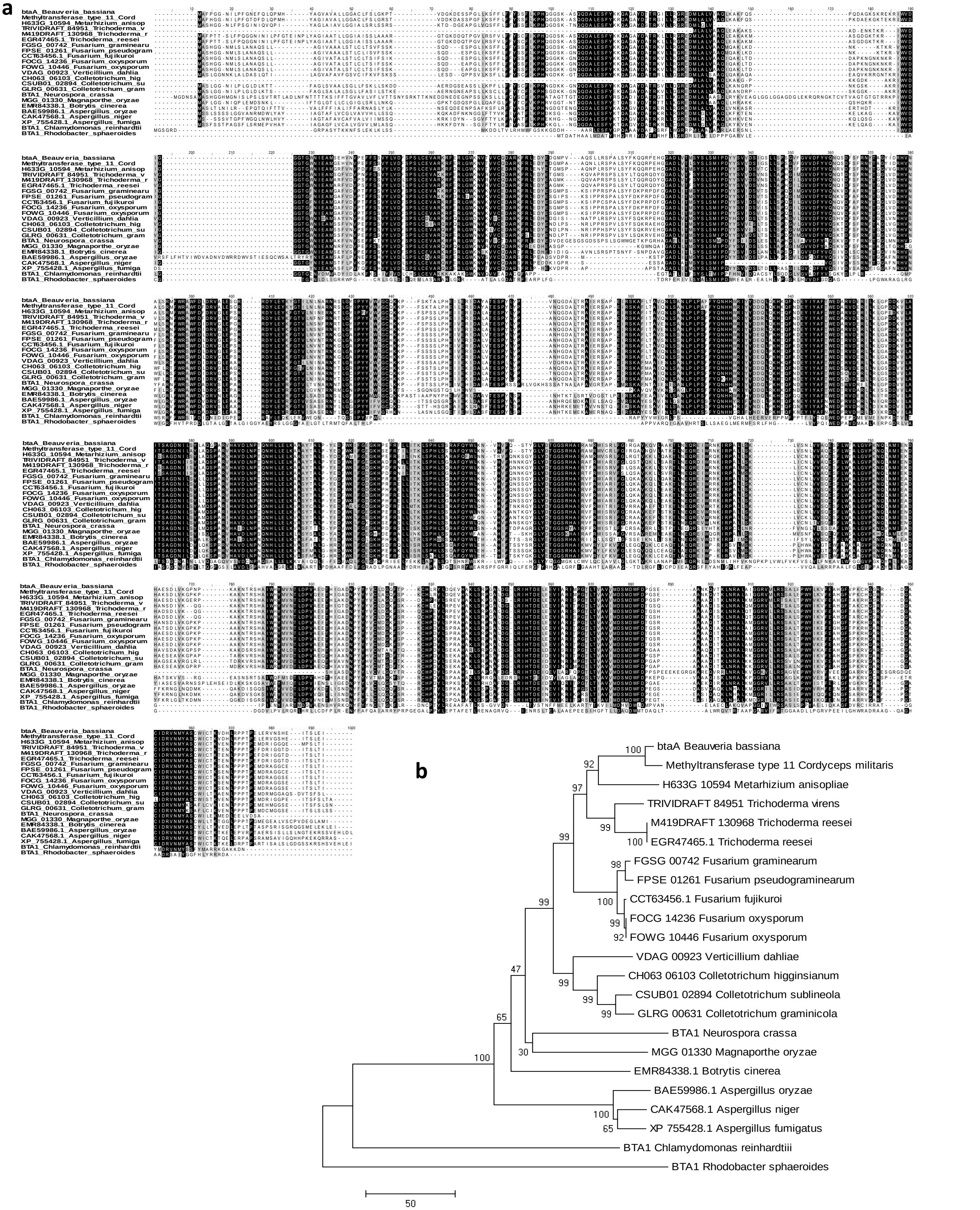

Figure P. Sequence alignment and phylogenetic comparison of BTA1.
The BTA1 gene homologs from 23 different species are analyzed. **(a)** Sequence alignment using MEGA6. **(b)** The Neighbor-Joining phylogenetic tree produced by MEGA6 software based on a multiple alignment of ClustaW. Bootstrap analysis (1,000 replicates) confirmed the structure of the tree.

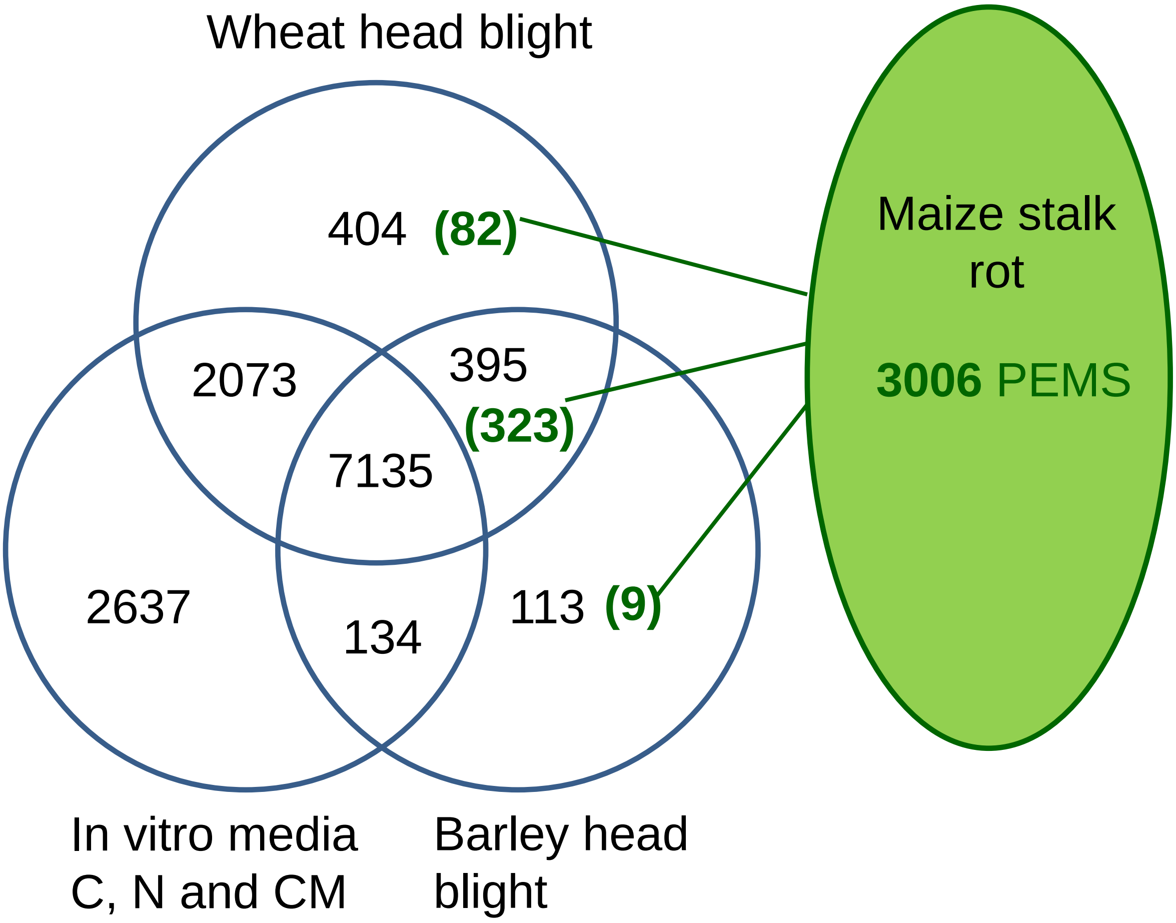

Figure Q. Comparison of *F. graminearum* gene expression in different hosts.
Comparison of 3,006 genes preferentially expressed during hyphal growth in maize stalk (PEMS) (this work) to microarray data from wheat and barley head blight infection (Lysoe et al., 2011, Fig 3)

Lysoe E, Seong KY, Kistler HC (2011) The Transcriptome of *Fusarium graminearum* During the Infection of Wheat. Mol Plant Microbe Interact 24: 995-1000.

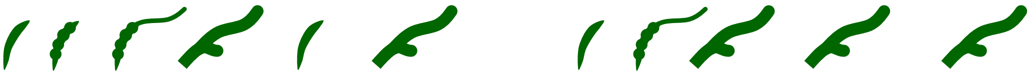

CFEM protein	*in vitro* (h)					in wheat coleptile (hai)					in maize stalk (hai)								
	0	2	8	24	72	0	16	40	64	240	0	12	18	36	48	72	108	132	144
FGSG_00588	9.9	8.1	9.4	9.7	7.8	8.2	8.3	7.3	6.7	7.3	8.2	8.1	9.3	8.8	8.4	7.2	8.7	6.8	7.5
FGSG_02077	11.5	7.7	10.3	12.6	6.7	7.8	10.3	11.6	10.0	7.8	7.8	7.7	10.0	7.7	9.6	8.2	8.4	8.1	9.0
FGSG_02109	2.2	2.6	2.6	2.4	2.6	2.5	2.6	2.6	2.7	2.5	2.5	2.5	2.6	2.5	2.5	2.5	2.6	2.7	2.6
FGSG_02155	4.5	3.3	3.3	3.0	3.3	4.3	6.5	5.5	6.7	5.4	4.3	7.7	6.4	5.3	4.4	6.9	10.3	8.0	7.0
FGSG_02181	4.0	4.4	4.3	3.9	4.1	4.0	4.5	4.4	3.9	3.2	4.0	4.0	4.3	3.9	4.1	4.0	3.3	4.4	3.8
FGSG_02840	2.7	3.9	7.7	8.3	10.8	4.0	8.6	5.0	7.8	4.7	4.0	8.1	7.5	6.6	6.1	5.6	5.0	3.4	3.1
FGSG_03573	3.7	3.8	3.8	3.7	4.2	4.2	3.9	4.0	3.8	3.5	4.2	4.0	4.4	3.8	4.0	3.9	3.7	3.9	3.9
FGSG_03599	3.8	3.9	4.0	3.6	3.6	3.6	4.9	4.1	10.9	9.9	3.6	4.1	4.7	3.6	6.5	6.8	6.3	6.9	8.3
FGSG_03897	6.7	8.1	4.9	3.2	5.6	9.0	3.3	3.4	2.6	5.7	9.0	2.8	3.1	3.3	3.2	4.5	4.4	5.2	3.2
FGSG_04529	2.9	6.9	4.3	3.8	7.3	4.5	4.2	3.7	3.1	4.5	4.5	4.8	5.3	4.8	6.0	4.6	3.5	3.8	3.1
FGSG_05175	7.3	8.4	7.9	12.1	8.3	7.2	10.4	10.7	9.6	8.1	7.2	7.2	8.3	8.5	8.3	9.2	6.9	8.9	6.4
FGSG_05821	2.7	2.7	2.6	2.8	2.9	3.0	2.7	3.1	2.7	3.4	3.0	2.8	2.7	3.3	2.9	3.6	2.7	3.1	2.5
FGSG_07839	2.6	3.8	3.0	2.6	3.9	3.7	3.0	3.3	3.0	5.9	3.7	3.0	3.0	4.9	2.9	3.3	3.1	2.9	2.9
FGSG_08554	9.8	6.9	8.1	8.4	4.8	8.9	6.7	5.1	6.4	6.6	8.9	6.6	7.1	6.8	8.1	6.4	7.6	6.6	9.8
FGSG_08844	4.5	5.1	6.9	9.0	7.2	5.0	7.8	6.7	7.6	7.4	5.0	8.8	7.9	10.6	6.3	7.3	7.5	7.3	7.9
FGSG_09675	3.1	3.0	3.1	3.1	3.3	2.9	3.2	3.3	3.0	2.8	2.9	3.3	2.9	3.1	3.2	2.9	2.9	3.0	3.1
FGSG_13972	5.6	4.1	2.7	2.7	2.8	4.2	3.1	3.2	3.1	3.2	4.2	3.3	3.1	2.9	3.2	2.9	3.0	3.6	3.3
FGSG_15975	2.7	2.9	3.0	2.8	4.0	3.0	3.0	3.0	2.9	3.9	3.0	3.1	2.9	3.1	3.0	3.0	3.0	3.2	2.9
FGSG_16221	2.5	2.6	2.7	2.6	2.6	2.7	2.8	3.0	2.8	2.7	2.7	2.9	3.1	2.7	2.5	2.9	2.6	2.7	2.5
FGSG_16254	2.8	2.9	2.9	2.8	3.0	3.1	3.8	3.5	4.1	3.3	3.1	3.0	3.1	3.0	2.9	3.1	2.9	3.4	2.8
FGSG_16423	9.6	6.6	3.1	2.6	2.5	9.7	3.8	2.7	3.3	6.4	9.7	2.7	2.8	2.7	2.5	2.9	2.4	2.6	2.6

Figure R. Expression of *F. graminearum* CFEM genes *in vitro*, in wheat coleoptile and in maize stalk.
The heat maps of eight-cysteine-containing fungal extracellular membrane (CFEM) protein genes show similarity in expression patterns during in vitro and *in planta* growth. The green icons on the top indicate the fungal morphology at the stages below. *In vitro* 0 to 24 h data from [81], wheat coleoptile data from [38], other data from this work.

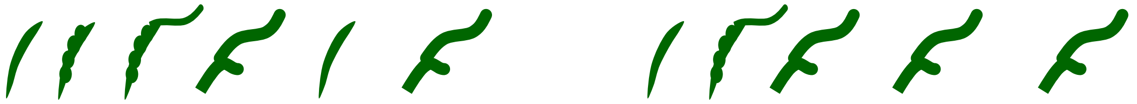

[SG]--P-C-[KR]-P motif proteins	in vitro (h)					in wheat coleptile (hai)					in maize stalk (hai)								
	0	2	8	24	72	0	16	40	64	240	0	12	18	36	48	72	108	132	144
FGSG_00129	2.8	3.2	7.2	3.0	2.8	3.1	5.9	5.0	3.3	3.3	3.1	8.1	7.7	8.0	6.3	7.9	8.4	5.5	3.7
FGSG_00131	2.5	2.5	2.6	2.6	2.4	2.4	2.5	2.6	2.4	2.4	2.4	2.6	2.7	2.3	2.4	2.5	2.3	2.6	2.6
FGSG_01595	2.9	3.3	6.7	6.6	2.3	3.7	5.5	3.9	2.5	4.3	3.7	7.5	5.1	6.4	6.4	6.0	6.9	4.3	3.3
FGSG_01598	3.0	3.2	3.5	3.2	3.1	3.2	3.2	3.1	3.1	2.8	3.2	3.3	2.9	3.1	2.9	3.2	2.8	3.1	3.0
FGSG_02036	3.2	4.2	4.0	3.7	3.8	3.1	6.5	4.9	3.8	5.4	3.1	4.9	4.5	5.4	3.5	7.4	6.9	4.4	3.8
FGSG_02154	4.7	3.8	5.5	4.1	3.3	3.9	6.4	5.2	4.7	4.1	3.9	7.2	5.5	6.3	4.3	8.1	8.5	6.8	4.5
FGSG_02269	4.4	4.2	6.6	5.8	2.7	3.3	7.4	6.4	6.2	5.3	3.3	7.5	6.8	8.5	5.5	8.6	9.5	8.2	6.6
FGSG_02888	2.4	2.8	2.9	2.6	5.4	2.7	3.9	2.8	2.9	2.6	2.7	6.1	2.7	5.1	2.4	3.8	2.5	2.8	2.5
FGSG_02899	2.8	3.1	5.1	5.8	3.4	3.4	4.6	3.7	3.7	3.8	3.4	5.8	4.0	4.4	3.4	3.9	3.1	3.4	3.2
FGSG_02913	2.7	3.0	7.7	3.2	2.8	2.9	4.9	3.6	2.9	2.7	2.9	6.5	6.0	5.0	4.9	4.2	4.6	3.2	3.2
FGSG_02999	3.5	3.5	7.5	4.9	4.6	4.6	5.7	5.8	3.9	4.0	4.6	7.3	5.9	8.0	5.5	7.4	8.1	5.1	4.1
FGSG_04429	4.0	6.6	7.8	6.3	3.6	6.9	7.7	7.0	5.1	6.8	6.9	7.7	5.8	8.3	4.3	7.4	8.2	6.0	6.5
FGSG_04521	2.6	2.6	3.3	2.5	2.8	3.2	4.8	3.6	3.1	2.9	3.2	4.5	2.9	4.0	3.0	4.7	4.3	3.0	3.0
FGSG_04687	2.4	2.4	2.4	2.3	2.2	2.4	2.4	2.7	2.3	2.3	2.4	2.3	2.2	2.2	2.3	2.5	2.5	2.5	2.4
FGSG_04739	3.3	3.3	6.5	3.2	3.8	4.0	6.4	4.5	3.2	3.4	4.0	8.1	5.2	9.4	6.8	5.5	4.5	3.3	3.3
FGSG_04740	3.0	4.3	6.2	3.0	2.9	3.0	4.7	3.4	3.0	3.8	3.0	5.1	4.4	5.2	3.5	5.2	4.7	3.0	3.3
FGSG_04841	3.5	7.3	8.1	4.8	3.2	5.9	7.4	6.1	4.4	6.9	5.9	8.9	7.9	9.1	6.9	9.0	9.5	8.3	7.3
FGSG_04842	2.5	3.1	6.4	2.5	2.6	2.7	5.3	4.2	2.8	2.8	2.7	7.4	4.3	6.4	5.4	5.1	4.5	2.9	2.4
FGSG_04858	3.1	3.5	6.3	4.6	3.6	3.7	7.2	5.9	4.2	5.2	3.7	8.5	7.6	7.9	6.0	7.9	8.3	7.0	5.5
FGSG_05947	2.8	5.9	7.1	2.4	2.7	4.1	7.1	4.7	3.2	5.8	4.1	8.2	4.9	7.6	4.8	8.8	9.7	7.8	5.7
FGSG_07435	3.1	3.7	4.4	3.7	3.0	3.4	2.9	3.1	2.9	2.9	3.4	3.3	2.8	2.9	2.9	3.0	2.6	3.1	3.0
FGSG_07522	5.8	9.7	11.6	10.1	6.5	6.5	8.3	7.6	3.6	6.7	6.5	8.0	9.5	8.3	7.0	7.2	5.7	4.9	4.8
FGSG_07629	2.5	5.3	5.5	2.9	3.0	3.9	7.6	4.6	4.0	6.5	3.9	7.7	5.7	6.3	5.4	7.1	7.8	5.7	4.1
FGSG_07684	3.0	3.9	4.1	3.3	3.4	4.8	6.0	4.5	4.1	3.5	4.8	6.0	3.9	6.0	3.6	7.8	8.7	6.9	6.0
FGSG_08094	2.8	3.2	4.7	2.9	2.8	2.9	5.9	3.6	3.0	3.8	2.9	8.0	4.0	7.4	3.4	7.5	8.3	5.9	3.1
FGSG_08095	2.6	2.6	2.6	2.6	2.4	2.5	2.7	2.5	2.5	2.5	2.5	2.6	2.4	2.7	2.5	3.0	2.5	3.0	2.5
FGSG_10495	6.3	5.3	5.3	5.0	5.9	6.5	5.8	5.3	4.5	4.2	6.5	6.3	4.7	7.2	4.7	8.4	8.2	5.8	5.0
FGSG_11136	2.7	2.8	3.1	2.7	2.6	2.9	4.0	3.1	2.7	2.9	2.9	4.8	3.0	3.6	2.8	4.0	3.2	3.0	2.7
FGSG_11156	3.3	4.0	5.0	3.4	5.6	3.6	4.7	4.1	3.6	3.3	3.6	6.0	4.1	4.6	3.5	5.0	4.7	3.8	3.4
FGSG_11166	3.8	4.0	8.5	7.8	3.7	4.5	7.6	5.5	4.1	7.1	4.5	9.2	6.5	8.8	5.6	8.4	8.9	7.3	5.7
FGSG_11238	3.5	3.7	4.4	2.7	2.9	4.0	5.3	4.7	2.8	3.7	4.0	7.5	5.6	6.6	4.3	7.8	5.8	4.4	3.0
FGSG_11418	2.4	2.2	2.4	2.3	2.1	2.3	2.3	2.4	2.4	2.1	2.3	3.6	2.4	2.5	2.2	2.5	3.5	2.3	2.1
FGSG_11540	3.5	7.4	9.8	10.0	3.5	4.4	8.5	7.7	5.7	7.2	4.4	10.4	9.2	10.6	6.8	9.8	10.0	7.3	6.4
FGSG_11715	2.4	2.6	2.6	2.4	2.4	2.7	3.0	2.8	2.6	2.3	2.7	3.2	2.7	2.6	2.5	2.7	2.6	2.6	2.6
FGSG_12107	2.8	2.9	2.9	2.7	2.8	2.6	3.0	3.0	2.6	2.5	2.6	3.3	3.0	3.0	2.7	2.8	2.7	2.8	2.7
FGSG_12405	2.6	2.8	3.1	2.6	2.5	3.2	4.4	3.8	2.8	2.6	3.2	4.1	4.1	4.4	3.1	5.3	4.2	2.9	2.7
FGSG_12434	4.9	6.3	7.1	6.7	5.7	5.1	6.1	6.0	4.3	5.2	5.1	8.9	7.4	8.8	5.6	9.0	7.8	6.3	4.7
FGSG_12486	2.5	2.6	2.7	2.5	2.8	2.7	3.0	3.0	2.8	2.7	2.7	2.7	3.0	2.8	2.8	2.6	2.5	2.9	2.7
FGSG_12591	7.2	3.4	5.5	3.0	2.8	3.3	5.6	3.9	3.7	4.3	3.3	8.1	5.0	7.8	4.7	7.3	7.7	4.7	5.1
FGSG_12644	2.9	3.3	4.5	3.1	3.4	3.7	5.5	4.1	3.5	3.5	3.7	6.7	5.1	5.6	4.3	6.8	5.8	4.7	3.6
FGSG_13692	2.3	2.2	2.5	2.4	2.6	2.5	2.7	2.6	2.4	2.6	2.5	3.2	2.5	2.7	2.4	2.9	3.5	2.9	2.5
FGSG_15679	2.7	2.6	2.6	2.7	2.7	2.7	2.8	2.7	2.9	2.8	2.7	3.1	2.8	2.7	2.8	2.9	2.7	2.8	2.9
FGSG_16104	2.5	2.8	4.5	2.8	2.7	3.7	4.9	3.5	2.9	3.2	3.7	4.7	3.2	3.4	2.9	4.0	3.4	2.5	2.8
FGSG_16431	3.4	3.7	6.4	3.8	3.5	3.7	5.5	4.9	3.9	3.5	3.7	6.7	4.6	6.3	4.1	6.1	5.5	4.3	3.8
FGSG_17419	2.6	2.6	3.2	2.6	2.5	2.6	3.5	2.8	2.5	2.4	2.6	4.6	3.0	2.8	2.6	2.8	2.9	2.6	2.7
FGSG_17425	2.6	3.8	6.8	3.8	3.5	3.2	6.0	3.7	2.9	3.5	3.2	7.7	4.5	7.9	3.8	7.6	7.5	4.8	3.2

Figure S. Expression of *F. graminearum* [SG]-P-C-[KR]-P motif protein genes *in vitro*, in wheat coleoptile and in maize stalk. The heat maps represent the expression of the fungal cell surface [SG]-P-C-[KR]-P motif protein genes which are similar during *in vitro* or *in planta* growth. Values indicate log_2 transformed intensities after global RMA normalization. The green icons on the top indicate the fungal morphology at the stages below. *In vitro* 0 to 24 h data from [81], wheat coleoptile data from [38], other data from this work.

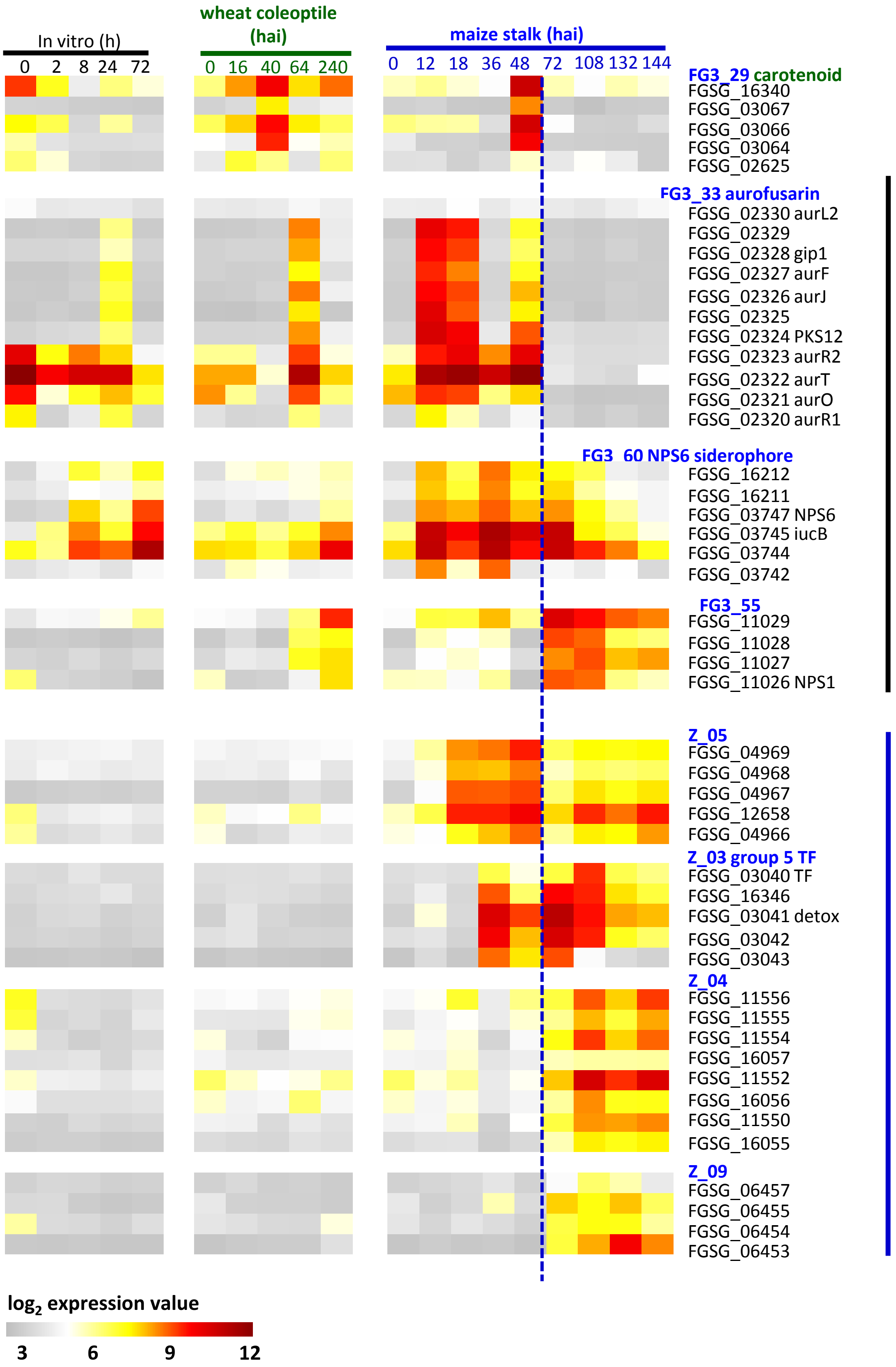

Figure T. Expression of *F. graminearum* SMB cluster genes *in vitro*, in wheat coleoptile and in maize stalk. The heat maps represent the co-expression of SMB cluster genes. Note that expression of some clusters are different between wheat and maize infection. *In vitro* 0 to 24 h data from [81], wheat coleoptile data from [38], other data from this work

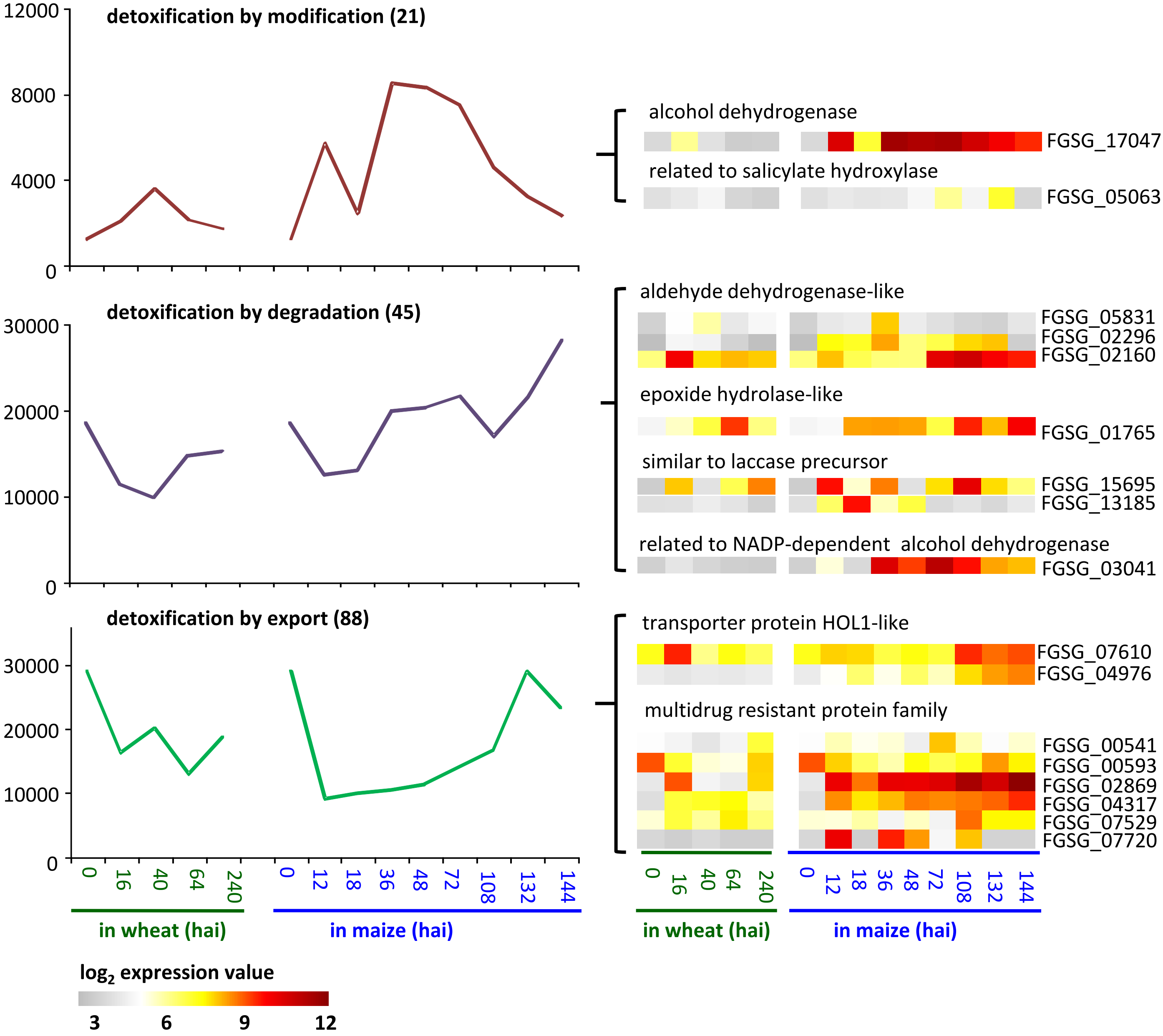

Figure U. Expression of *F. graminearum* detoxification-related genes *in vitro*, in wheat coleoptile and in maize stalk. Note that expression of some genes are different between wheat and maize infection. Y-axis: total intensities of all genes in the denoted group. Wheat data from [38], maize data from this work

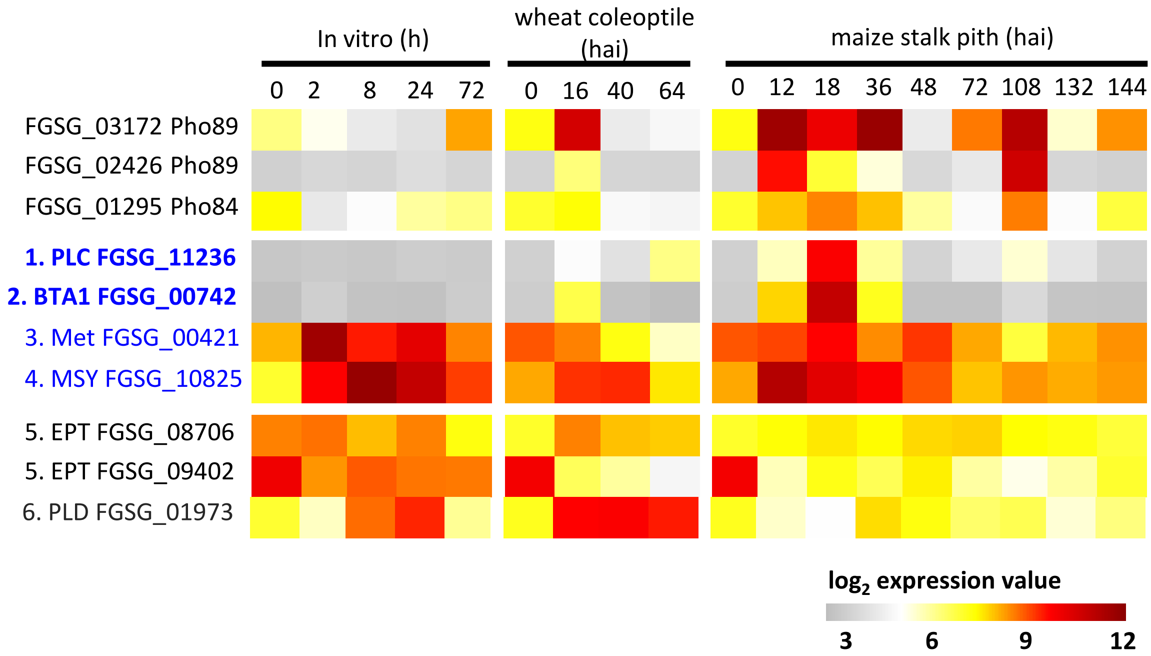

Figure V. Expression of *F. graminearum* genes related to membrane lipid metabolism *in vitro*, in wheat coleoptile and in maize stalk. *In vitro* 0 to 24 h data from [81], wheat coleoptile data from [38], other data from this work.

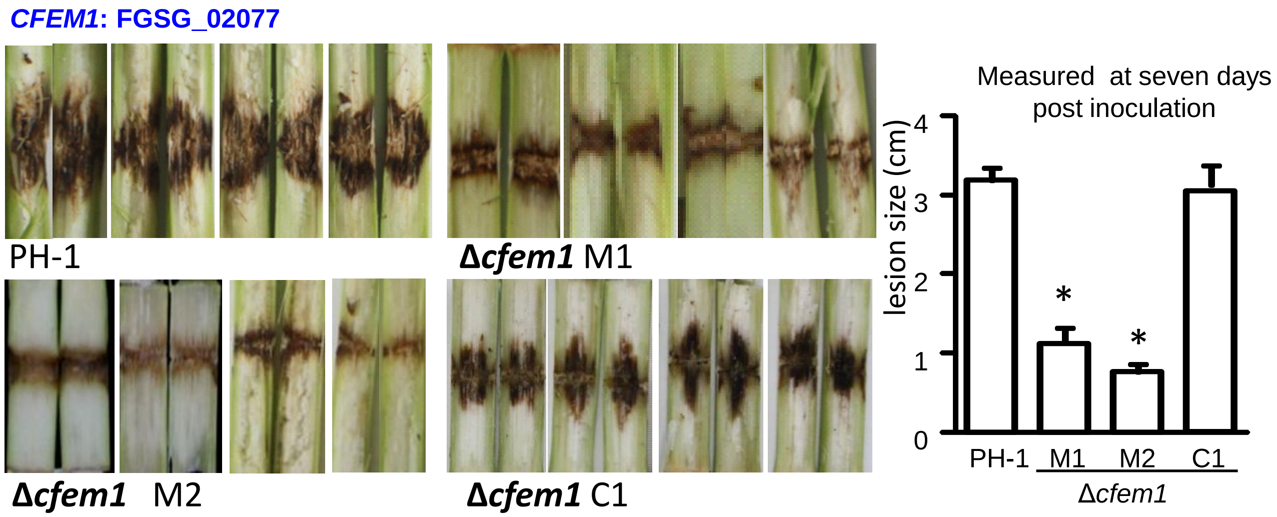

Figure W. Maize stalk virulence assay for CFEM1 related strains.
Lesions were measured at seven days after inoculation. * Significantly different from wild type (P < 0.05) according to Student's *t* test, n = 3 independent experiments. Error bars denote SE. C1 represents complemented strain of M1.

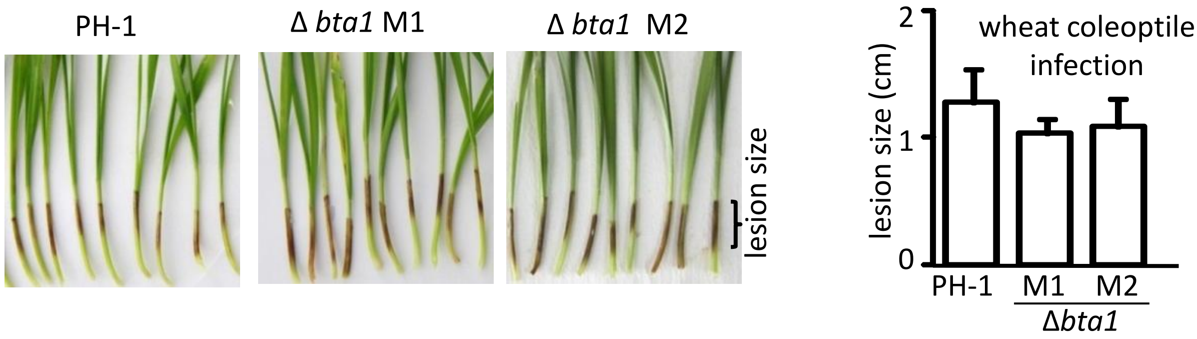

Figure X. Virulence on wheat coleoptiles of *Δbta1* mutants. Lesions were measured at seven days after inoculation. No significant difference between wild type and mutant caused lesion (P < 0.05) according to Student's *t* test, n = 3 independent experiments.

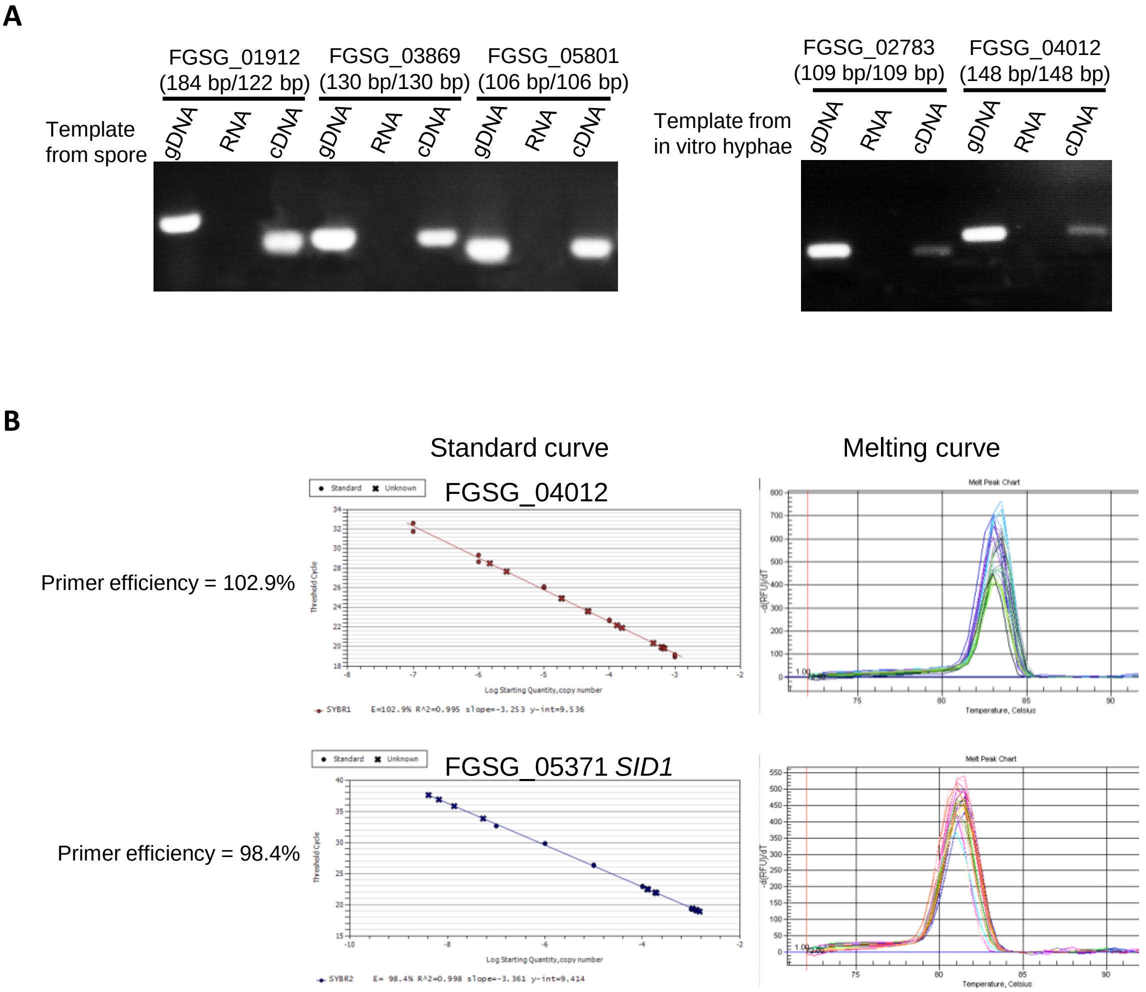

Figure Y. Quality control for template RNAs and primers used in quantitative RT-PCR.
(A) PCR products using genomic DNA (gDNA), RNA or cDNA (complementary DNA) as templates. The RNA templates extracted from spore or *in vitro* grown hyphae were used for quantitative RT-PCR analysis (in this work). cDNA were derived from these RNA templates after reverse transcription. Expected PCR product sizes from gDNA and cDNA are provided in parentheses below. The results show that no PCR products can be amplified from RNA templates without reverse transcription for the tested genes, indicating no genomic DNA contamination in our RNA templates. (B) Serial dilution standard curves and melting curves for two representative primer pairs used in our quantitative RT-PCR. Results show that primer efficiencies are in the range 95%-105%, and only one specific product peak was amplified for each reaction.
